# Tumor-targeted Gd-doped mesoporous Fe_3_O_4_ nanoparticles for T_1_/T_2_ MR imaging guided synergistic cancer therapy

**DOI:** 10.1080/10717544.2021.1909177

**Published:** 2021-04-19

**Authors:** Shaohui Zheng, Shang Jin, Min Jiao, Wenjun Wang, Xiaoyu Zhou, Jie Xu, Yong Wang, Peipei Dou, Zhen Jin, Changyu Wu, Jingjing Li, Xinting Ge, Kai Xu

**Affiliations:** a School of Medical Imaging, Xuzhou Medical University, Xuzhou, China; b Department of Radiology, Affiliated Hospital of Xuzhou Medical University, Xuzhou, China; c Institute of Medical Imaging and Digital Medicine, Xuzhou Medical University, Xuzhou, China; d College of Medical Engineering, Xinxiang Key Laboratory of Neurobiosensor, Xinxiang Medical University, Xinxiang, Henan , China; e School of Information Science and Engineering, Shandong Normal University, Jinan, China

**Keywords:** Tumor targeting, Gd doping, mesoporous Fe_3_O_4_ NPs, T1/T2 MR imaging, synergistic cancer therapy

## Abstract

In this study, a novel intelligent nanoplatform to integrate multiple imaging and therapeutic functions for targeted cancer theranostics. The nanoplatform, DOX@Gd-MFe_3_O_4_ NPs, was constructed Gd-doped mesoporous Fe_3_O_4_ nanoparticles following with the doxorubicin (DOX) loading in the mesopores of the NPs. The DOX@Gd-MFe_3_O_4_ NPs exhibited good properties in colloidal dispersity, photothermal conversion, NIR triggered drug release, and high T_1_/T_2_ relaxicity rate (*r*_1_=9.64 mM^−1^s^−1^, *r*_2_= 177.71 mM^−1^s^−1^). Benefiting from the high MR contrast, DOX@Gd-MFe_3_O_4_ NPs enabled simultaneous T_1_/T_2_ dual-modal MR imagining on 4T1 bearing mice *in vivo* and the MR contrast effect was further strengthened by external magnetic field. In addition, the DOX@Gd-MFe_3_O_4_ NPs revealed the strongest inhibition to the growth of 4T1 *in vitro* and *in vivo* under NIR irradiation and guidance of external magnetic field. Moreover, biosafety was also validated by *in vitro* and *in vivo* tests. Thus, the prepared DOX@Gd-MFe_3_O_4_ NPs would provide a promising intelligent nanoplatform for dual-modal MR imagining guided synergistic therapy in cancer theranostics.

## 1. Introduction

Despite the advance of modern medical technology, cancer remains as a great threat to human health (Torre et al., 2016). Clinically, the conventional therapies, such as surgery, radiotherapy, and chemotherapy still cannot effectively conquer this disease ascribed to their low therapeutic efficacy and severe adverse effects (Vanneman & Dranoff, 2012; Gotwals et al., 2017; Lee et al., 2018). Fortunately, nanoparticle-based strategies have emerged in the development of alternative cancer therapies owing to their advantages for overcoming the limitations associated with the conventional treatments (Perez-Herrero & Fernandez-Medarde, 2015). More recently, multifunctional nanoparticles have attracted more attention owing to their versatility that can integrate multiple functions such as molecular imaging, chemotherapeutics, active tumor targeting, phototherapy, and immunotherapy, into one nanoplatform. These intelligent nanomaterials would provide a novel strategy in nanomedicine for cancer theranostics (Arranja et al., 2017; Liu et al., 2018).

Among these versatile functions, medical imaging occupies the primary role in precise cancer diagnosis, which is also able to guide the cancer treatment process (Kijima et al., 2014). Up to date, various imaging methods have been developed for biomedical imaging, including fluorescence imaging (FL), magnetic resonance imaging (MRI), computed tomography imaging (CT), position emission tomography (PET), photoacoustic imaging (PA), etc. (Cuevas & Shibata, 2009; Berges et al., 2010; Fan et al., 2014; Tempany et al., 2015). Among the various medical imaging techniques, magnetic resonance imaging (MRI) is a very powerful and noninvasive imaging tool to provide high 3D spatially resolved images with the information on the anatomy, function and metabolism of tissues *in vivo* (Fernando et al., 2013; Lu et al., 2013). Normally, MR imaging is performed in T_1_ or T_2_ mode based on the T_1_-weighted contrast agents (CAs) or T_2_-weighted CAs, respectively, to improve the sensitivity of MR imaging. However, each contrast agent possesses its own merits and limitations in MR imaging applications (Czeyda-Pommersheim et al., 2017).

Clinically, Gd^3+^-based paramagnetic complexes, such as Gd-DTPA, are widely used in T_1_-weighted MR imaging to obtain brighter images. The T_1_-weighted MR enhancement is realized by reducing the longitudinal relaxation times to provide positive contrast (Courant et al., 2012; Gupta et al., 2015; Phukan et al., 2018). However, these Gd^3+^ complexes are always prepared into small molecules, which would lead to fast clearance from body and hamper the further engineering of the complexes (Boehm & Heverhagen, 2018). On the other side, superparamagnetic iron oxide nanoparticles (SPIONs) are extensively investigated as T_2_ Cas (Qin et al., 2015). These SPIONs are able to reduce transverse relaxations times to produce darker images and negative contrast in the T_2_-weighted MR imaging practice (Cheng et al., 2011, 2012). Unfortunately, for the low signal body regions, it would be difficult to distinguish the damaged tissues with hemorrhages, calcification, fat, blood clots and other possible artifacts, which would hamper the further potential in MR imaging. Thus, the development of dual-modal T_1_–T_2_ CAs would provide superior contrast effect in both T_1_ and T_2_-weighted MR images to conquer problems associated with the CAs with signal modality (Yang et al., 2011; Zhou et al., 2017; Li et al., 2018).

Recently, photothermal therapy (PTT) induced by near infrared (NIR) laser has become a promising therapeutic strategy to destroy tumor in a noninvasive way (Hou et al., 2018; Liu et al., 2019). Normally, the photothermal tumor ablation can be achieved by using the laser absorbing agents to convert laser energy into thermal energy locally at tumor site. Till date, a variety of nanomaterials have been developed as laser absorbing agents, such as some gold nanoparticles, carbon-based nanomaterials, and semiconductor nanostructures (Robinson et al., 2010; You et al., 2012; Huang et al., 2017). Particularly, besides the MRI practice as T_2_ CAs, the iron oxide nanoparticles (Fe_3_O_4_ NPs) also exhibit good photothermal converting ability for PTT in cancer. For example, Shen et al. successfully prepared individual magnetic Fe_3_O_4_ NPs and applied them in photothermal therapy with NIR irradiation (Shen et al., 2015). Zhou et al. also proposed iron/iron oxide core/shell nanoparticles for magnetic targeting MRI and near-infrared photothermal therapy (Zhou et al., 2014).

**Scheme 1.**
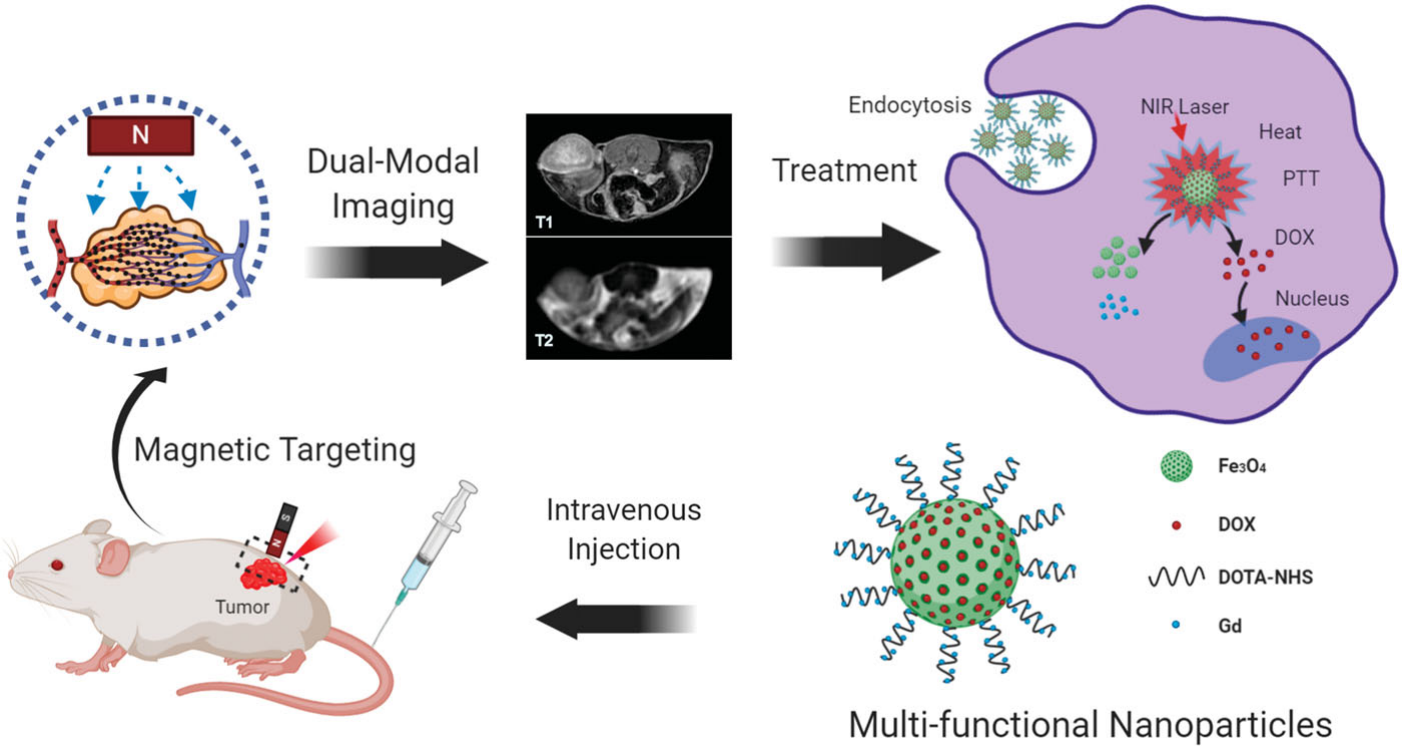
Schematic illustration of the preparation of DOX@Gd-MFe_3_O_4_ NPs and magnetic tumor targeted T_1_/T_2_ MR imaging guided synergistic cancer therapy.

Herein, as shown in Scheme 1, we propose a novel multifunctional nanoplatform for magnetic targeted T_1_–T_2_ dual MR imaging guided combined cancer therapy. The nanoplatform (DOX@Gd-MFe_3_O_4_ NPs) was constructed by Gd-doped mesoporous Fe_3_O_4_ nanoparticles following with the doxorubicin (DOX) loading in the mesopores of the NPs. The prepared DOX@Gd-MFe_3_O_4_ NPs exhibited good colloidal dispersity, superior magnetization, high r_1_ and r_2_ relaxavity, excellent photothermal conversion, and NIR-triggered drug release. The *in vitro* cellular uptake was investigated by confocal laser scanning microscope and Prussian blue staining, which the influence of NIR laser and external magnetic field was also explored. Benefiting from the outcome of photothermal conversion and cellular uptake, the *in vitro* combined PTT and chemotherapy was evaluated by live/dead cell staining and MTT assay. Next, we moved to investigate the magnetic targeted T_1_/T_2_-weighted MR imaging on 4T1 tumor bearing mice *in vivo*. Furthermore, the synergistic PTT and chemotherapy under the guidance of MRI was also verified on 4T1 tumor bearing mice *in vivo*. Finally, the long-term biosafety of the Gd-MFe_3_O_4_ NPs was explored *in vitro* and *in vivo*. Thus, we expect the prepared DOX@Gd-MFe_3_O_4_ NPs would favor tumor targeted drug delivery for T_1_/T_2_ dual modal MR imaging guided combined tumor therapy.

## 2. Materials and methods

### 2.1. Materials

Iron(III) chloride hexahydrate (FeCl_3_·6H_2_O), ethylene glycol (EG), ethylenediamine (ETH), gadolinium chloride hexahydrate(GdCl_3_·6H_2_O), doxorubicin hydrochloride (DOX·HCl), and sodium acetate anhydrous (NaAc) were purchased from Sinopharm Chemical Reagents Co. Ltd (Shanghai, China). 1,4,7,10-Tetraazacyclododecane-1,4,7,10-tetraacetic acid mono-*N*-hydroxysuccinimide ester (DOTA-NHS-ester), 1-(3-dimethylaminopropyl)-3-ethylcarbodiimide hydrochloride (EDC), poly(methacrylic acid sodium salt) (PMAA) and MTT assay were received from Sigma-Aldrich Co. (St. Louis, MO). Prussian blue iron stain kit (with nuclear fast red solution) was obtained from Beijing Solarbio Science & Technology Co. Ltd (Beijing, China). Dulbecco’s Modified Eagle Medium (DMEM), fetal bovine serum (FBS), PBS buffer, EDTA-trypsin, and penicillin/streptomycin were received from Gibco BRL (Gaithersburg, MD). All other chemicals used in this study were of analytical grade.

### 2.2. Synthesis of DOX loaded Gd-doped mesoporous Fe_3_O_4_ nanoparticles (DOX@Gd-MFe_3_O_4_ NPs)

#### 2.2.1. Synthesis of Gd-doped mesoporous Fe_3_O_4_ NPs

Mesoporous Fe_3_O_4_ (MFe_3_O_4_) NPs were synthesized by previously reported hydrothermal method (Guo et al., 2009). FeCl_3_·6H_2_O (1 g) was first dissolved in ethylene glycol (20 mL) to form a clear solution. Then, NaAc (3 g) and ethanediamine (10 mL) were added into the above solution to form a homogenous mixture under 30 min vigorous stirring. Afterwards, the resultant mixture was sealed into a teflonlined stainless-steel autoclave, heated and maintained at 200 °C for 8 h. Subsequently, the product was naturally cooled down to ambient temperature and washed carefully with DI water. Thereafter, the prepared MFe_3_O_4_ NPs were further carboxylated with PMAA. MFe_3_O_4_ NPs (30 mg) were dispersed in 50 mL DI water, following with the addition of 3 mL PMAA solution (30% wt). Then, the mixture was sonicated for 1 h and then maintained overnight for complete carboxylation of the surface. Afterwards, the product was washed several times to remove the unreacted PMAA.

Subsequently, Gd was attached on MFe_3_O_4_ NPs by the chelation with DOTA. Carboxylated MFe_3_O_4_ NPs (3 mg) were dispersed in 3 mL DI water, and then EDC (30 mg) and DOTA-NHS (3 mL, 12 mM) were added under stirring for 6 h. Thereafter, the product was washed thrice with DI water to remove excess EDC and DOTA-NHS. Next, DOTA-MFe_3_O_4_ NPs (5 mg) were dispersed in 5 mL GdCl3 solution (12 mM) and maintained for 12 h for the Gd chelation. Finally, the product was centrifuged and washed to obtained the Gd-MFe_3_O_4_ NPs. In addition, the content of Gd and Fe element in the Gd-MFe_3_O_4_ NPs were measured by an inductively coupled plasma-mass spectrometry (ICP-MS).

#### 2.2.2. DOX loading in Gd-MFe_3_O_4_ NPs

Free DOX (3 mg) was firstly dissolved in 1 mL PBS buffer and mixed with above Gd-MFe_3_O_4_ NPs (2 mg/mL, 5 mL). Then, the resultant mixture was stirred at 500 rpm overnight in the dark and then centrifuged at 10,000 rpm for 10 min. Afterwards, the NPs were washed thrice with DI water to remove the unloaded DOX and the supernatant solution was collected to investigate the loading efficacy. Finally, the DOX@Gd-MFe_3_O_4_ NPs were centrifuged, washed, and freeze-dried for the further use. The drug loading efficiency (LE) and encapsulation efficiency (EE) can be calculated according to following equations:

$$\mathrm{LE}\;(\%)=\frac{W_{\mathrm{initial}}-W_{\mathrm{remanent}}}{W_{Gd-\mathrm{MFe}3O4}}\times 100\%;\text{ EE }(\%)\;=\frac{W_{\mathrm{initial}}-W_{\mathrm{remanent}}}{W_{\mathrm{initial}}}\times 100\%$$

*W*_initial_ is the weight of DOX in initial solution, *W*_remanent_ is the weight of DOX in supernatant, and Gd-MFe_3_O_4_ is the weight of Gd-MFe_3_O_4_ NPs added in solution.

### 2.3. Characterization of DOX@Gd-Fe_3_O_4_ NPs

The shape and morphology of DOX@Gd-Fe_3_O_4_ NPs was studied by scanning electron microscopy (SEM, SU8010, Hitachi, Tokyo, Japan) and transmission electron microscopy (TEM, FEI Tecnai G2 Spirit). The particle size distribution and surface zeta potentials of DOX@Gd-Fe_3_O_4_ NPs were investigated by a Zetasizer Nano ZS90 (Malvern Instruments Ltd, Malvern, UK). The chemical structure of DOX@Gd-Fe_3_O_4_ NPs was explored using a Fourier Transform Infrared spectrometer (FTIR, Bruker Tensor27, Billerica, MA). N_2_ adsorption-desorption isotherms were assessed using a surface area and pore characterization system (ASAP 2020, AutoChem II). UV–vis–NIR absorption spectra was determined by an UV–vis–NIR spectrometer. X-ray diffraction (XRD) measurements was carried out by a high-resolution X-ray diffractometer (XRD, X’ Pert PRO Multi-Purpose, XRD). The magnetization was evaluated by vibrating sample magnetometer (VSM, Lake Shore Cryotronics 7404, Westerville, OH) at 300 K.

### 2.4. Photothermal conversion test of DOX@Gd-MFe_3_O_4_ NPs

The photothermal conversion test was explored on 0.5 mL DOX@Gd-MFe_3_O_4_ NPs suspension under 808 nm laser irradiation (FC-808-10W, Changchun New Industries Optoelectronics Technology Co. Ltd, Changchun, China). The DOX@Gd-MFe_3_O_4_ NPs were suspended in PBS buffer at various concentrations (PBS, 0.05, 0,10, 0.25, 0.50, and 1.00 mg/mL) and placed in a quartz cuvette. Then, the suspensions were irradiated by NIR laser at different intensities (0.2, 0.5, 1, 1.5, 2 W/cm^2^) for 5 min. An IR thermal imaging camera (FLIR E60; FLIR Systems, Inc., Wilsonville, OR) was deployed to monitor the temperature elevation of the solutions. Furthermore, we investigated the photothermal stability by repeatedly exposing the DOX@Gd-MFe_3_O_4_ NPs suspension to NIR laser (laser on) and then naturally cooled to room temperature (laser off) for 4 cycles.

### 2.5. *In vitro* DOX release profile from DOX@Gd-MFe_3_O_4_ NPs

The *in vitro* release of DOX from DOX@Gd-MFe_3_O_4_ NPs was performed using the standard dialysis method in the presence and absence of NIR laser. Briefly, DOX@Gd-MFe_3_O_4_ NPs were suspended in 2 mL PBS buffer (containing 2 mg DOX), transferred in a dialysis bag (MWCO: 3500 Da), and then placed in a tube containing 50 mL PBS buffer. Thereafter, 1 mL samples were withdrawn at pre-determined time points and replaced by 1 mL fresh PBS buffer. Simultaneously, NIR laser (2 W/cm^2^) was used to trigger the release at 4 and 12 h time point. The concentration of released DOX in each sample was analyzed by a microplate reader.

### 2.6. *In vitro* cell studies

#### 2.6.1. Cell culture

4T1 mouse breast cancer cells (4T1) and NIH3T3 mouse embryonic fibroblast cells (HSF) were obtained from American Type Culture Collection (ATCC, Manassas, VA) and maintained in RPMI medium containing 10% (v/v) FBS and 1% 1% penicillin–streptomycin. The cells were incubated at 37 °C in a humidified 5% CO_2_ atmosphere.

#### 2.6.2. In vitro cell uptake study

The *in vitro* cell uptake of the DOX@Gd-MFe_3_O_4_ NPs was first studied by the confocal laser scanning microscopy (CLSM). Briefly, 4T1 cells were seeded on sterile coverslips in a 12-well plate (3 × 10^4^ cells/well) for 24 h to allow cell attachment. Then, DOX@Gd-MFe_3_O_4_ NPs (DOX= 5 μg/mL, NPs = 48 μg/mL) were added in each well and co-incubated with cells for 2 h in the presence and absence of a magnet (magnetic field: 0.05 T). Simultaneously, NIR laser (1.5 W/cm^2^, 5 min) was used to irradiate the cells during the NPs incubation to explore the effect of NIR laser to the intracellular drug release. Thereafter, the cells were washed thrice with PBS solution and fixed with 4% PFA. Then, the cells were mounted with gold anti-fade mounting medium containing DAPI and observed with a CLSM (CLSM, LSM880, Zeiss, Jena, Germany).

The cellular uptake of DOX@Gd-MFe_3_O_4_ NPs was further performed by the Prussian blue staining to label the distribution of Fe inside tumor cells. 4T1 cells were cultured on Φ35 mm petri dishes for 24 h and then treated with DOX@Gd-MFe_3_O_4_ NPs (100 μg/mL and 200 μg/mL) for 4 h with and without a magnet. Then, the cells were washed carefully with PBS buffer and fixed with 4% PFA solution. Subsequently, the cells were subjected to Prussian blue staining for 15 min and nuclear fast red staining for 5 min, respectively. Finally, the samples were observed by an optical microscopy to image the distribution of Fe inside cells.

#### 2.6.3. *In vitro* combined therapy of DOX@Gd-Fe_3_O_4_ NPs

The *in vitro* combined therapeutic performance was firstly evaluated on 4T1 cells by live/dead cell staining. 4T1 cells were seed on Φ35 mm petri dishes overnight and then treated with DOX, Gd-MFe_3_O_4_ NPs and DOX@Gd-MFe_3_O_4_ NPs (0.2 mg/mL) in the presence and absence of magnetic field. Afterwards, the cells were rinsed cleanly and exposed to NIR laser (1.5 W/cm^2^) for 5 min. Subsequently, cells were stained with calcein AM and PI to label the live and dead cells after various treatments. Finally, the samples were imaged by a fluorescent microscope.

Afterwards, the synergistic anticancer effect of DOX@Gd-MFe_3_O_4_ NPs was further quantitively assessed by the MTT assay. 4T1 cells were seed in 96-well plate overnight. Then, DOX, Gd-MFe_3_O_4_ NPs and DOX@Gd-MFe_3_O_4_ NPs of various concentrations were separately added to each well and co-incubated with cells with and without a magnet for 24 h. Thereafter, some groups of the cells were subjected to NIR irradiation (1.5 W/cm^2^) for 5 min and further incubated for another 24 h. Finally, the MTT assay was conducted to evaluate the surviving cells in each well. The cell viability could be calculated as the percentage of surviving cells as means of triplicate tests.

### 2.7. Magnetic resonance imaging performance

#### 2.7.1. T1/T_2_ relaxivity evaluation

The T*_1_*/T*_2_*-weighted relaxation studies of DOX@Gd-MFe_3_O_4_ NPs were conducted on a 3.0 T GE Discovery 750 W MR scanner. The DOX@Gd-MFe_3_O_4_ NPs were dispersed in 0.5% agarose gel at various Gd/Fe concentrations. The T_1_ and T_2_ phantom images and relaxation time were obtained separately using the spin-echo sequence. The parameters for T_1_-weighted MRI could be set as: TR= 425 ms, TE = Min Full, matrix size = 384 × 224, field of view= 18 cm × 18 cm, slice thickness= 3.0 mm, and spacing = 1.5 mm. The T_2_-weighted MR phantom images and relaxation times were acquired according to following parameters: repetition time (TR) = 3000 ms, echo time (TE) = 10 ms.

#### 2.7.2. *In vivo* MR tumor imaging

Balb/c mice were supplied by the Animal Center of Xuzhou Medical University and the animal studies were approved by the Animal Care Committee of Xuzhou Medical University. For the tumor model development, 4T1 cells (2 × 10^6^ cells in 100 µL) were subcutaneously injected on the back of each mouse. Thereafter, after the tumor grew to about 100 mm^3^, the mice were intravenously injected with DOX@Gd-MFe_3_O_4_ NPs in with and without a magnet at the tumor site during the experiment. Afterwards, T*_1_*/T*_2_*-weighted MR imaging studies were carried out at the determined time points (0, 1 h, 2 h, 3 h, 4 h, 8 h, 12 h, and 24 h) on the previous MR scanner with a special coil designed for small animal imaging.

### 2.8. *In vivo* combined antitumor activity

4T1 tumor-bearing Balb/c mice were randomly separated into seven groups to evaluate the therapeutic outcome of combined PTT and chemotherapy: (i) saline, (ii) NIR, (iii) DOX@Gd-MFe_3_O_4_ NPs, (iv) DOX, (v) Gd-MFe_3_O_4_ NPs + NIR, (vi) DOX@Gd-MFe_3_O_4_ NPs + NIR, and (vii) DOX@Gd-MFe_3_O_4_ NPs + NIR + magnet (10 mg/kg). The mice were injected with the above formulations via tail vein and then subjected to NIR laser irradiation (2 W/cm^2^, 5 min) after 24 h injection for the NIR-treated groups. The temperature change of the tumor site was recorded by the previous IR thermal imaging system. During the treatment period, tumor volume and body weight were monitored every other day for 3 weeks. The tumor volume could be calculated according to the equation as: V = (length of tumor) × (width of tumor)^2^/2.

### 2.9. *In vivo* biosafety study

The long-term biosafety study was conducted on healthy Balb/c mice for a 21-d period. Balb/c mice were (7 weeks) were randomly separated into two groups and injected with saline and Gd-MFe_3_O_4_ NPs (10 mg/kg). Blood samples were collected at 0, 1, 7, and 21-d after injection from the mice for blood routine and biochemistry analysis. Afterwards, major organs were harvested, fixed with 10% formalin, and subjected to histological analysis. The tissue histological samples were imaged using a microscope.

### 2.10. Statistical analysis

Student’s *t*-tests were used to assess the statistical significance between two groups. The *p* value less than .05 was considered statistically significant. All results in this work were presented as mean value ± standard deviation.

## 3. Results and discussions

### 3.1. Characterization of DOX@Gd-MFe_3_O_4_ NPs

**Figure 1.**
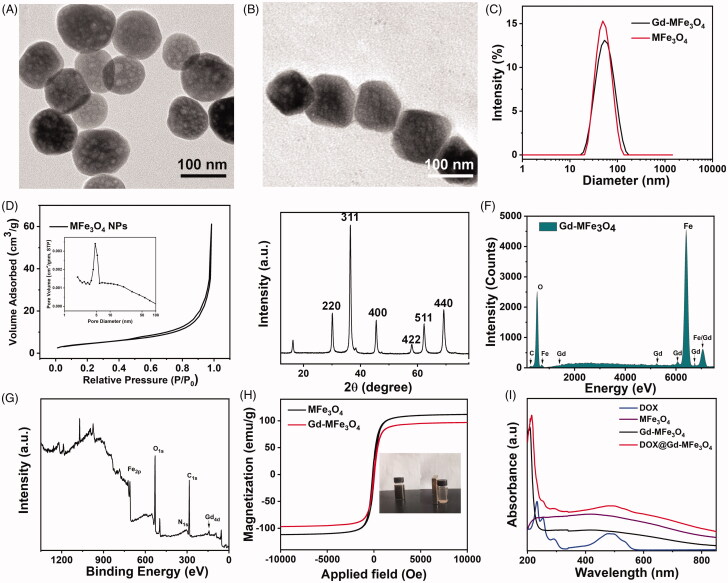
Characterizations of Gd-MFe_3_O_4_ NPs: (A) TEM image of MFe_3_O_4_ NPs; (B) TEM image of Gd-MFe_3_O_4_ NPs; (C) size distribution of MFe_3_O_4_ and Gd-MFe_3_O_4_ NPs; (D) nitrogen adsorption–desorption isotherm of the MFe_3_O_4_ NPs; (E) XRD pattern of MFe_3_O_4_ NPs; (F) EDS analysis of Gd-MFe_3_O_4_ NPs; (G) XPS spectra of Gd-MFe_3_O_4_ NPs; (H) magnetization curve of the MFe_3_O_4_ and Gd-MFe_3_O_4_ NPs; (I) UV–-vis–NIR absorbance spectra of DOX, MFe_3_O_4,_ Gd-MFe_3_O_4,_ DOX@Gd-MFe_3_O_4_ NPs.

In this study, DOX@Gd-MFe_3_O_4_ NPs were constructed by Gd-doped mesoporous Fe_3_O_4_ nanoparticles following with the doxorubicin (DOX) loading in the mesopores of the NPs for magnetic targeted T_1_–T_2_ dual MR imaging guided combined cancer therapy. First, mesoporous Fe_3_O_4_ NPs were synthesized by the hydrothermal method. The size and the morphology of MFe_3_O_4_ NPs were explored by TEM as shown in Figure 1(A). The MFe_3_O_4_ NPs exhibited uniform spherical shape with an average diameter of about 49 nm and clear mesoporous nanostructure (Figure 1(A,C)). BET analysis was also deployed to investigate the mesoporous structures of the MFe_3_O_4_ NPs. From the nitrogen adsorption–desorption in Figure 1(D), the surface area of the was evaluated to be 12.3 m^2^g^−1^, which was enough for the drug encapsulation. The pore size analysis indicated the size distribution in the range of 3.52–5.87 nm with a peak pore size of 4. 7 nm. The crystal structure of the MFe_3_O_4_ NPs was explored by the X-ray diffraction (XRD) analysis. As shown in Figure 1(E), the synthesized MFe_3_O_4_ NPs revealed the X-ray diffraction peaks ascribed to the 220, 311, 400, 422, 511, and 440 planes. The XRD peaks matched well with the standard peaks for the magnetite (JCPDS 89-4319), indicating the successful synthesis of MFe_3_O_4_ NPs.

Subsequently, DOX@Gd-MFe_3_O_4_ NPs were prepared by doping Gd on MFe_3_O_4_ NPs following with the doxorubicin (DOX) loading in the mesopores. The average size of DOX@Gd-MFe_3_O_4_ NPs increased slightly to 53 nm after the Gd and DOX modification. The energy-dispersive spectroscopy (EDS) pattern exhibited the presence of Fe, O, Gd, and C within the DOX@Gd-MFe_3_O_4_ NPs (Figure 1(F)). The X-ray photoelectron spectroscopy (XPS) analysis also revealed characteristic peaks of Fe (2p), O(1s), and Gd (4d) elements (Figures 1(G) and Figure S1). Figure 1(H) reveals that DOX@Gd-MFe_3_O_4_ NPs could be readily suspended in DI water to form a stable black dispersion in an aqueous solution. And the DOX@Gd-MFe_3_O_4_ NPs could be drawn to sidewall under an external magnet. The magnetic properties of MFe_3_O_4_ NPs were evaluated by a vibrating sample magnetometer (VSM) and analyzed as 112 emu/g, indicating their high magnetization of MFe_3_O_4_ NPs. In addition, the magnetization of DOX@Gd-MFe_3_O_4_ NPs slightly decreased to 98 emu/g attributed to the existence of non-magnetic components in the DOX@Gd-MFe_3_O_4_ NPs. In addition, we also investigated the UV–vis–NIR absorbance of these nanomaterials. As shown in Figure 1(I), the MFe_3_O_4_ NPs exhibited broad and continuous absorbed spectra in the NIR range. After DOX loading, a clear absorbance peak at 480 nm corresponding to DOX appeared in the absorbance of DOX@Gd-MFe_3_O_4_ NPs. Moreover, the loading efficiency of DOX in MFe_3_O_4_ NPs was explored and calculated to 10.5%. Thus, these results confirmed the successful synthesis of DOX@Gd-MFe_3_O_4_ NPs from multiple perspectives.

### 3.2. Photothermal effect of DOX@Gd-MFe_3_O_4_ NPs

**Figure 2.**
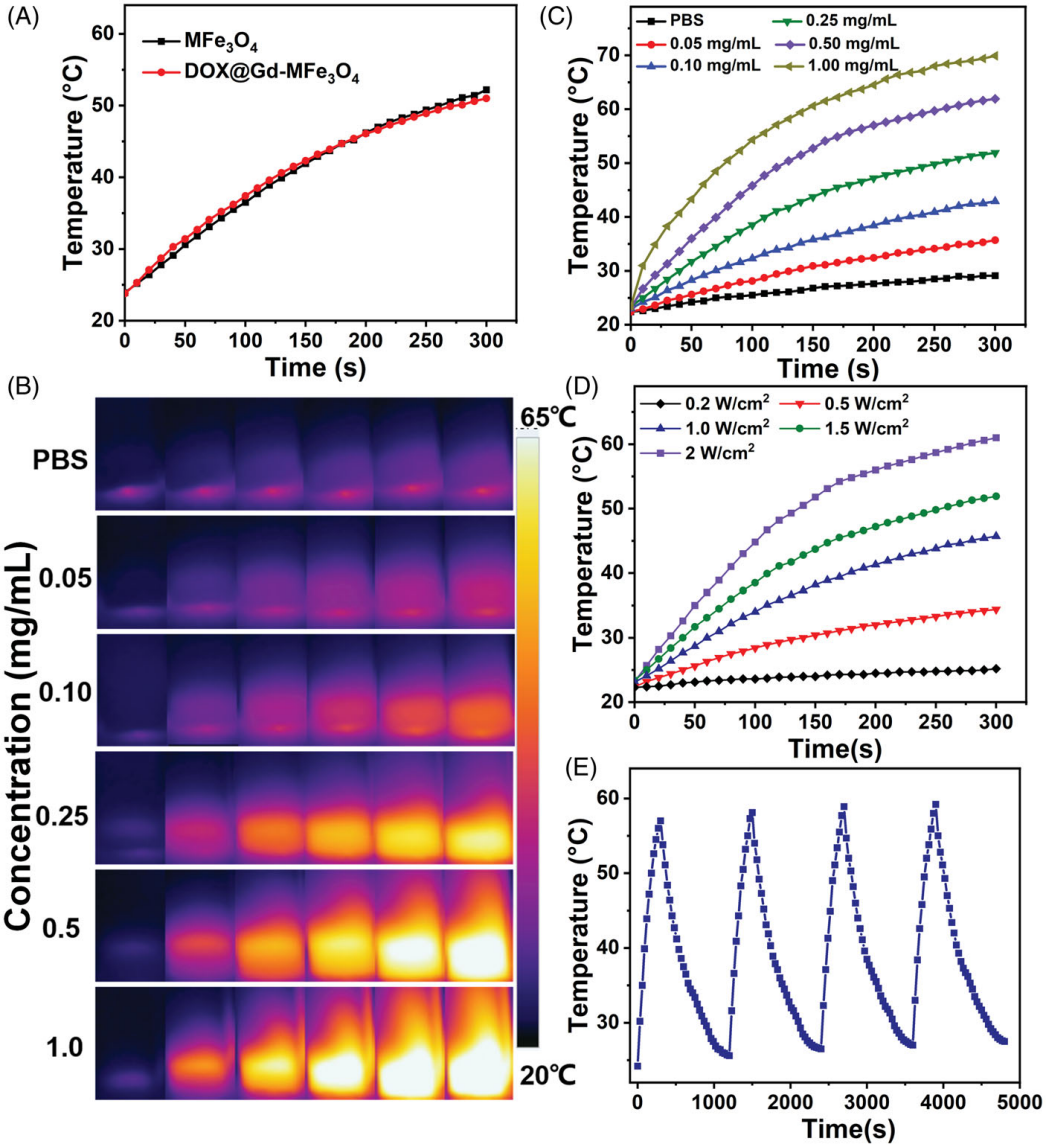
Photothermal effect of Gd-MFe_3_O_4_ NPs: (A) temperature change in MFe_3_O_4_ and Gd-MFe_3_O_4_ NPs suspension at the same concentration (0.25 mg/mL) under NIR laser irradiation (5 min, 1.5 W/cm^2^); (B) infrared thermal images of Gd-MFe_3_O_4_ NPs suspension of varying concentrations exposed to NIR laser (1.8 W/cm^2^) for 0–5 min. (C) Temperature elevation in Gd-MFe_3_O_4_ NPs suspension at gradient concentrations NIR laser irradiation (5 min, 1.5 W/cm^2^). (D) Temperature elevation in Gd-MFe_3_O_4_ NPs suspension (0.25 mg/mL) at different intensities of NIR laser irradiation. (E) Photothermal stability of Gd-MFe_3_O_4_ NPs suspension (0.25 mg/mL) under repeated NIR laser irradiation for four cycles.

The photothermal conversion is of great significance for the light-absorbing agent to be applied in PTT. The photothermal property of the DOX@Gd-MFe_3_O_4_ NPs with different concentrations was performed by exposing to NIR laser at various power intensities. As shown in Figure 2(A–C), upon the NIR irradiation, the nanoparticles solutions were rapidly heated to a plateau temperature and exhibited concentration-dependent temperature elevation in 5 min. The temperature of the particle solutions increased to from 35.7 °C to 69.9 °C as the concentrations ranged from 0.05 to 1 mg/mL. In contrast, the temperature of PBS only increased by 4.3 °C in the same condition. It demonstrated that the DOX@Gd-MFe_3_O_4_ NPs possessed good photothermal property to convert NIR laser into thermal energy. Subsequently, we further investigated the photothermal performance of DOX@Gd-MFe_3_O_4_ NPs under various NIR laser power intensities. As expected, as revealed in Figure 2(D), a greater NIR power density induced higher temperature elevation. The NPs solution could be easily heated to 45 °C at a low power density of 1 W/cm^2^, which could lead to irreversible damage to tumor cells. Moreover, the photothermal conversion efficiency of DOX@Gd-MFe_3_O_4_ NPs was measured and calculated to be 26.8% (Supplementary data). Furthermore, the photothermal conversion stability of DOX@Gd-MFe_3_O_4_ NPs was also investigated by repeatedly irradiating the DOX@Gd-MFe_3_O_4_ NPs solutions. As shown in Figure 2(E), the DOX@Gd-MFe_3_O_4_ NPs solutions exhibited identical performance in the four consecutive heating and cooling cycles, demonstrating the good photothermal stability of the DOX@Gd-MFe_3_O_4_ NPs. These results indicated the superior light-absorbing ability of DOX@Gd-MFe_3_O_4_ NPs for PTT application.

### 3.3. *In vitro* DOX release from DOX@Gd-MFe_3_O_4_ NPs

**Figure 3.**
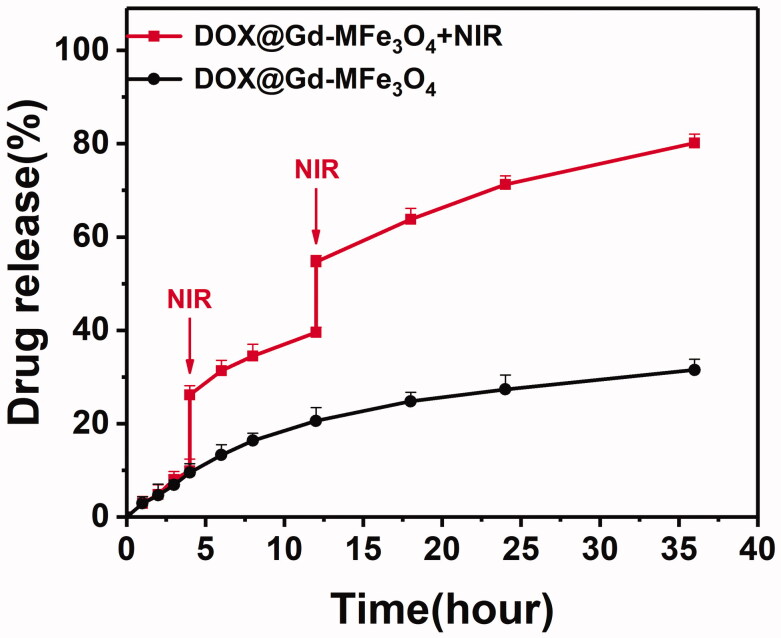
*In vitro* DOX release from DOX@Gd-MFe_3_O_4_ NPs in the presence and absence of NIR laser (2 W/cm^2^, 5 min). Values are expressed as mean ± SD (*n* = 3).

The drug release characteristics of DOX@Gd-MFe_3_O_4_ NPs were studied based on the fluorescence of DOX in the released medium by a dialysis method. In addition, we also investigated the influence of NIR irradiation on the DOX release. As shown in Figure 3, as a result, the DOX@Gd-MFe_3_O_4_ NPs exhibited relatively low release rate of 31%, attributed the holding effect of mesopore of MFe_3_O_4_ NPs. The small amount of DOX release might be attributed the leakage of DOX from MFe_3_O_4_ NPs. However, under NIR irradiation, the DOX release was significantly activated, where burst release behavior was observed shortly after NIR irradiation. Furthermore, the overall DOX release rate was accelerated under NIR irradiation. And at the end, almost 79% of DOX was liberated into the medium. It may be attributed to the local high temperature induced by the photothermal effect. These results demonstrated that NIR irradiation could be used to trigger the DOX release from DOX@Gd-MFe_3_O_4_ NPs to enable on-demand drug release specifically at tumor site.

### 3.4. T1 longitudinal and T_2_ transverse relaxivity

**Figure 4.**
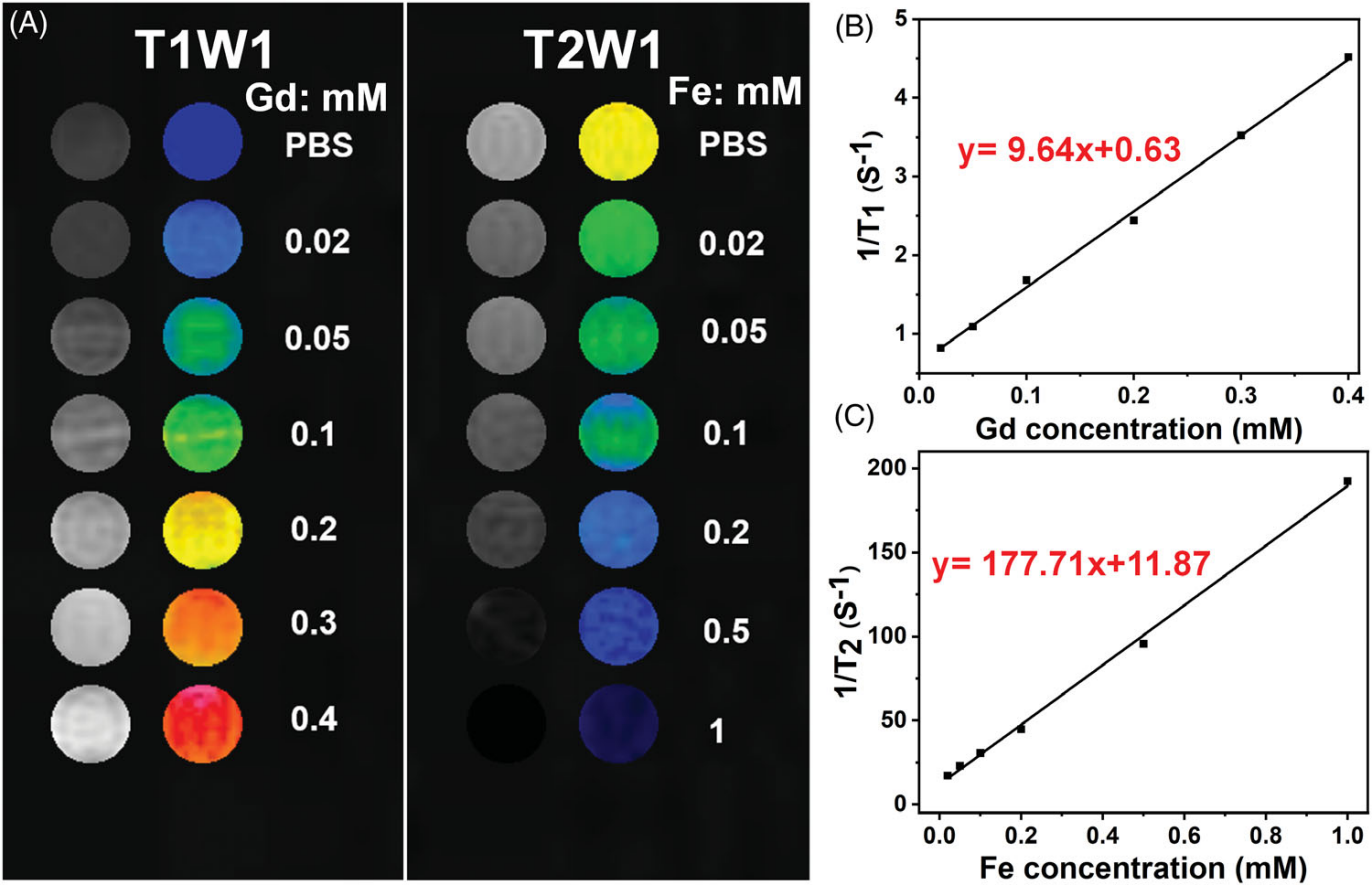
T_1_ longitudinal and T_2_ transverse relaxivities: (A) T_1_ and T_2_-weighted phantom images of Gd-MFe_3_O_4_ NPs using a 3.0 T MR scanner; (B) T_1_ longitudinal relaxation rate of Gd-MFe_3_O_4_ NPs with different concentrations of Gd; (C) T_2_ transverse relaxation rate of Gd-MFe_3_O_4_ NPs with different concentrations of Fe.

In this work, Gd was doped on the MFe_3_O_4_ NPs to form the Gd-MFe_3_O_4_ NPs for the simultaneous dual T_1_ and T_2_-weighted MR imaging. The longitudinal and transverse relaxivities of Gd-MFe_3_O_4_ NPs were investigated by the *in vitro* MR phantom tests at various concentrations dispersed in 0.5% agarose gel. As shown in Figure 4(A), the MR phantom images clearly revealed increasing brightening and darkening effect dependent on the increasing Gd and Fe concentrations, respectively. It confirmed the simultaneous T_1_- and T_2_-weighted dual contrast potential of Gd-MFe_3_O_4_ NPs in MR imaging. Furthermore, the T_1_ longitudinal and T_2_ transverse relaxivity was calculated to quantitatively evaluate the dual T_1_ and T_2_-weighted MR contrast ability of Gd-MFe_3_O_4_ NPs. Figure 4(B,C) shows the linear plots of longitudinal and transverse relaxation rates (r_1_ and r_2_) vs the concentrations of Gd and Fe in the samples, respectively. The Gd-MFe_3_O_4_ NPs revealed an enhanced r_1_ relaxivity of 9.64 mM^−1^s^−1^ compared to the commercial product Gd-DTPA of 4.45 mM^−1^s^−1^ (Li et al., 2018). On the other side, the r_2_ rate was found to be 177.71 mM^−1^s^−1^, which was much higher than many previous reports (Chen et al., 2011; Zhou et al., 2014). The high r_1_ and r_2_ rates would enable the Gd-MFe_3_O_4_ NPs in efficient dual T_1_ and T_2_-weighted MR imaging.

### 3.5. *In vitro* biocompatibility of Gd-MFe_3_O_4_ NPs

The biological safety is critical for nanomaterials to be used in biomedical fields, such as cancer diagnosis and therapy. The biocompatibility of Gd-MFe_3_O_4_ NPs was first evaluated *in vitro* on NIH3T3 and 4T1 cells by MTT assay, as shown in Figure S2. Both NIH3T3 and 4T1 cells exhibited good growth condition after the 24 h treatment of Gd-MFe_3_O_4_ NPs. And NIH3T3 and 4T1 cells remained high cell viabilities of 92% and 86%, respectively, at the highest concentration of 0.5 mg/mL. This result preliminary confirmed the biocompatibility of Gd-MFe_3_O_4_ NPs *in vitro*.

### 3.6. *In vitro* cellular uptake

**Figure 5.**
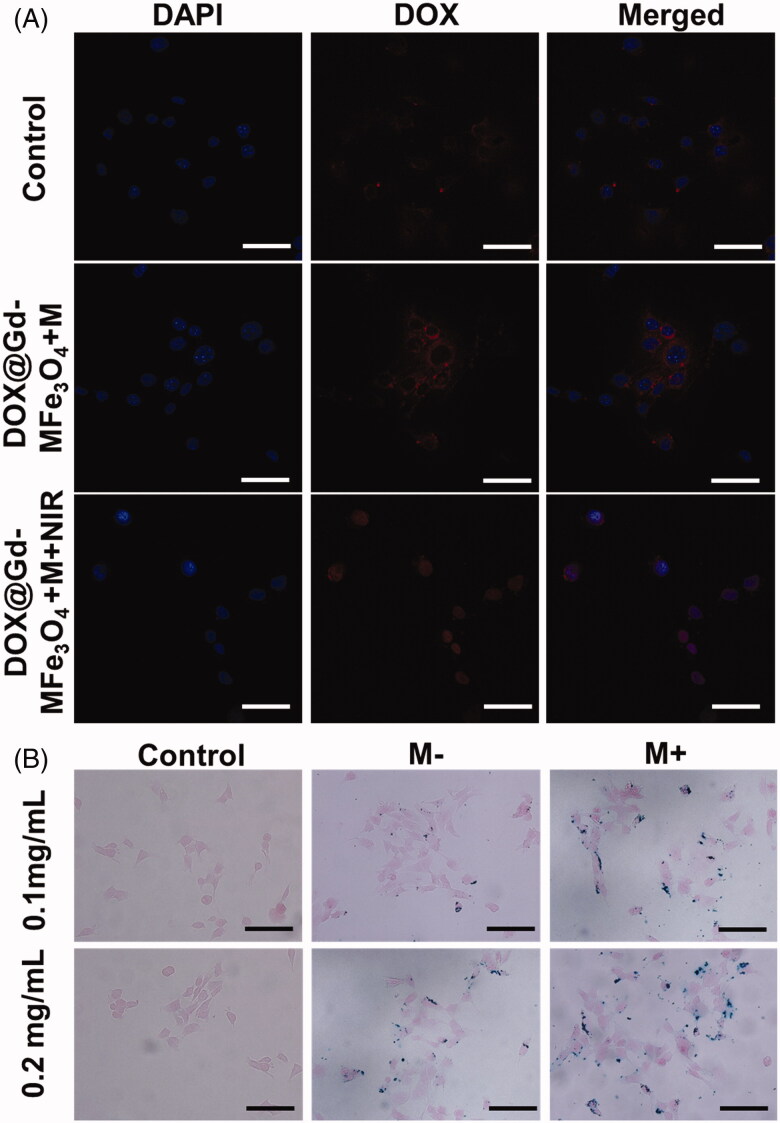
*In vitro* cellular uptake behavior of DOX@Gd-MFe_3_O_4_ NPs: (A) confocal microscopy images of the cellular uptake of DOX@Gd-MFe_3_O_4_ NPs (DOX= 5 μg/mL) 4T1 cells, scale bar = 20 μm. (B) Prussian blue staining of 4T1 cells in the presence and absence of magnetic field after 2 h incubation of Gd-MFe_3_O_4_ NPs with different concentrations (scale bar = 20 μm).

The cellular uptake efficacy is of great importance for the targeted intracellular delivery of nanomedicine for tumor therapy. In this study, cellular uptake was explored using CLSM and Prussian blue staining on 4T1 cells. In addition, we also investigated the influence of NIR irradiation and magnetic field to the internalization of DOX@Gd-MFe_3_O_4_ NPs. For CLSM observation, 4T1 cells were cultured on sterile coverslips and incubated with DOX@Gd-MFe_3_O_4_ NPs for 2 h with and without a magnet. A group of the samples was exposed to NIR laser (1.5 W/cm^2^, 5 min) for 5 min after 1 h incubation of the NPs. As shown in Figure 5(A), DOX@Gd-MFe_3_O_4_ NPs were engulfed inside 4T1 tumor cells with relatively low red fluorescence surrounding the blue cell nuclei. It may be attributed to low targeting ability and intracellular DOX release from DOX@Gd-MFe_3_O_4_ NPs. Subsequently, in the presence of magnetic field, the DOX signal was highly enhanced compared to the previous group, demonstrating a magnetic-field guided intracellular delivery of magnetic DOX@Gd-MFe_3_O_4_ NPs to tumor cells. Furthermore, we also deployed NIR laser to boost the tumor cellular uptake and trigger intracellular DOX release. After NIR irradiation, the DOX signal was much higher inside cells than the other groups. The result may be partly ascribed to the improved membrane permeability of tumor cells after NIR irradiation. The other reason is NIR laser could efficiently trigger the release of DOX from DOX@Gd-MFe_3_O_4_ NPs.

The behavior of internalization of DOX@Gd-MFe_3_O_4_ NPs was also measured by Prussian blue staining to directly determine the distribution of DOX@Gd-MFe_3_O_4_ NPs inside cells (Figure 5(B)). As a result, compared to the control groups, many blue spots were observed inside and attached to the tumor cells after co-incubation. In addition, much higher internalization of DOX@Gd-MFe_3_O_4_ NPs into cells was clearly confirmed under the guiding of magnetic field, which was consistent to the CLSM result. These results indicated the magnetic targeted tumor intracellular delivery of DOX@Gd-MFe_3_O_4_ NPs and NIR triggered release inside cells.

### 3.7. *In vitro* combined therapy of PTT and chemotherapy

**Figure 6.**
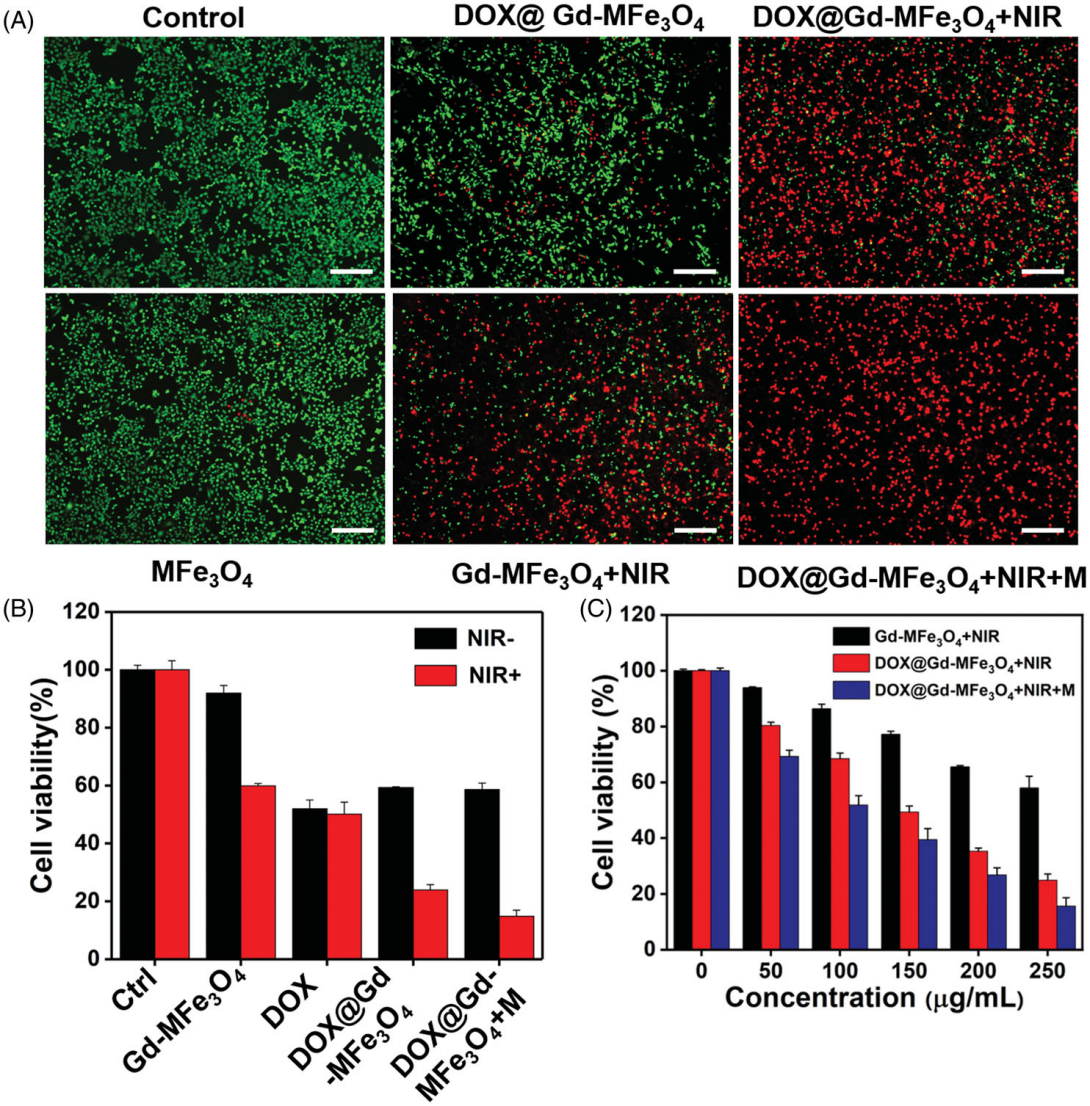
*In vitro* synergistic therapy for 4T1 tumor cells (A) Live/dead cells staining images of 4T1 cells after different treatments (0.25 mg/mL). The cells were co-stained with calcein AM and PI for live (green) and dead (red) cells, respectively. (B) Cell viabilities of 4T1 cells after different treatments with or without NIR. Values are expressed as mean ± SD (*n* = 4). (C) Cell viabilities of 4T1 cells after photothermal ablation with different NPs concentrations. Values are expressed as mean ± SD (*n* = 4).

Inspired by the superior photothermal performance of DOX@Gd-MFe_3_O_4_ NPs, we moved to investigate the anticancer performance of PTT combined with chemotherapy on 4T1 cells. Live/dead staining was first performed to evaluate the therapeutic outcome of combined therapy with Calcein AM and PI, and then observed by fluorescence microscope. As shown in Figure 6(A), the cells in control groups were not significantly influenced and remained good condition, suggesting Gd-MFe_3_O_4_ NPs possessed limited damage to tumor cells. For the DOX@Gd-MFe_3_O_4_ NPs group, a small part of cells was found to be dead owing to the released DOX inside tumor cells. In contrast, for the Gd-MFe_3_O_4_ NPs group upon NIR irradiation, cells were dramatically killed by the photothermal effect, indicating the excellent killing effect of the PTT only. Furthermore, when combined with DOX, the DOX@Gd-MFe_3_O_4_ NPs + NIR group exhibited even much more cells stained to be red with a few green fluorescence, suggesting the advantage of the combined therapy. Particularly, when we applied magnetic field to facilitate targeting of DOX@Gd-MFe_3_O_4_ NPs, all 4T1 cells were almost killed, indicating the therapeutic efficacy was remarkably enhanced by the improved cellular uptake mediated by external magnetic field.

The tumor therapeutic performance was further measured quantitatively by the MTT assay. As exhibited in Figure 6(B,C), NIR only and Gd-MFe_3_O_4_ NPs treated groups remained high cell viabilities above 90% compared to the control group. Free DOX induced similar killing effect of 54% to tumor cells with and without NIR laser. However, the DOX@Gd-MFe_3_O_4_ NPs groups with and without a magnet revealed limited anticancer effect due to the low release of DOX from DOX@Gd-MFe_3_O_4_ NPs inside tumor cells. However, under NIR irradiation, the cell viabilities of DOX@Gd-MFe_3_O_4_ NPs ± magnet were reduced to 13% and 24%, respectively, attributed to the synergistic effect of PTT and DOX. Besides, the cell viability was highly dependent on the concentration of different treatments of Gd-MFe_3_O_4_ NPs, DOX@Gd-MFe_3_O_4_ NPs, and DOX@Gd-MFe_3_O_4_ NPs + magnet under NIR irradiation. Especially, when the concentration increased to 0.25 mg/mL, cell viability of the DOX@Gd-MFe_3_O_4_ NPs + magnet group significantly decreased to 15.2%. These results suggested a good therapeutic outcome of the combined PTT and DOX therapy for cancer treatment.

### 3.8. *In vivo* magnetic targeted MR imaging

**Figure 7.**
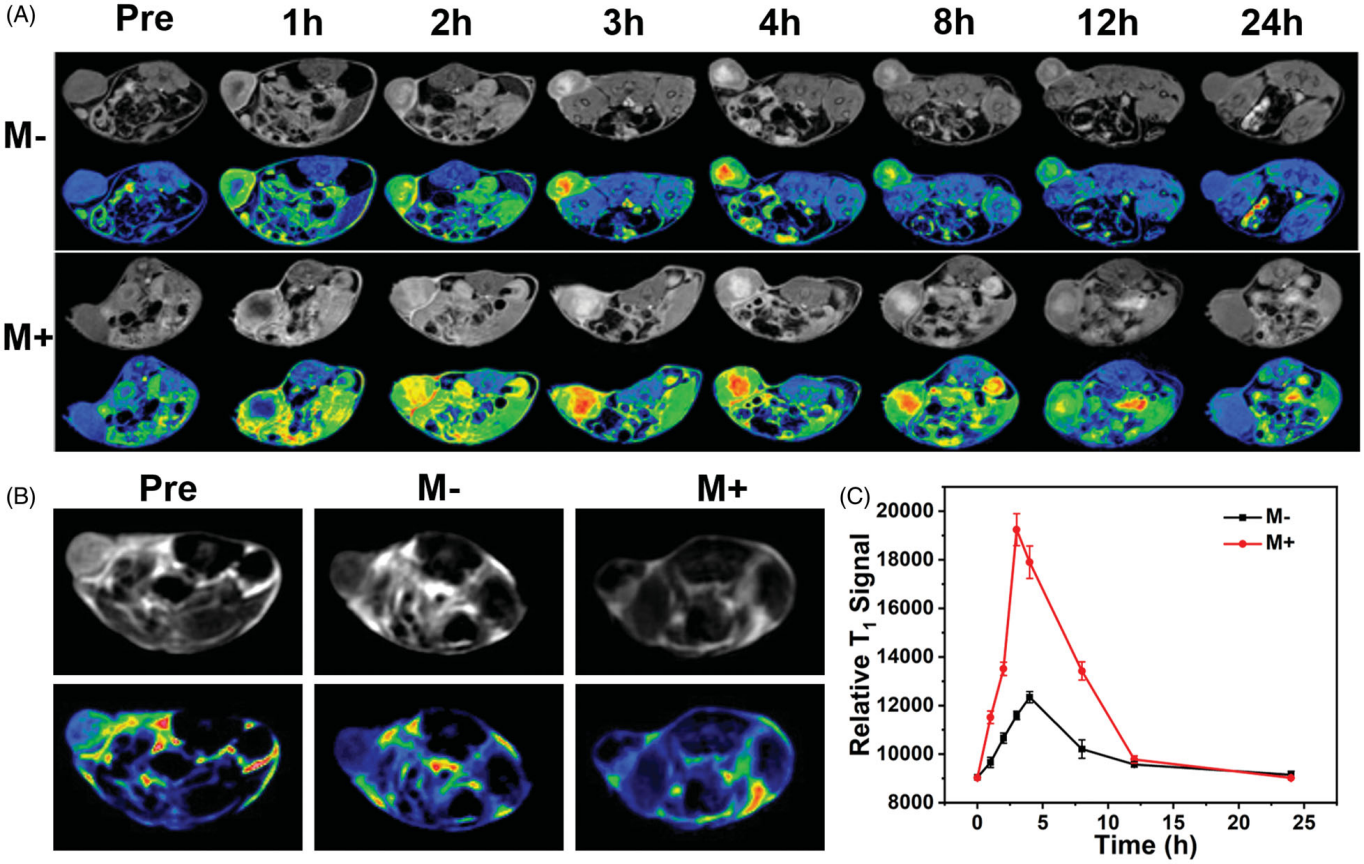
(A) *In vivo* MR imaging tests. (A) T_1_-weighted MR imaging of mice model with or without magnet after injection of Gd-MFe_3_O_4_ NPs at various time intervals; (B) T_2_-weighted MR imaging of mice model in the presence and absence of magnetic field at 4 h after injection of Gd-MFe_3_O_4_ NPs (C) T_1_-weighted MRI signals of the tumors at 0, 1, 2, 3, 4, 8, 12, and 24 h after administration of Gd-MFe_3_O_4_ NPs. Values are expressed as mean ± SD (*n* = 5).

Considering the high r_1_ and r_2_ relaxivity of the prepared DOX@Gd-MFe_3_O_4_ NPs, we further performed T_1_ and T_2_ weighted MR imaging on 4T1 bearing mice. The DOX@Gd-MFe_3_O_4_ NPs were administrated intravenously and then the mice were subjected to MR imaging at various time points. As shown in Figure 7(A), as expected, T_1_ signal of the tumor region became bright after 1 h injection and increased to the peak at 4 h. Thereafter, the T_1_ signal gradually decayed and almost disappeared after 24 h. To investigate the effect of magnetic field to the NPs accumulation, a magnet was also attached at the tumor site of each mouse of a group. As revealed in Figure 7(A), the MR signal was generally enhanced in all time points after magnet attachment. Especially, the MR intensity was extremely strengthened at 3 h and maintained a very high signal until 8 h compared to 1 h high intensity window period (3–4 h after injection) for the group without magnet attachment. It suggested that external magnetic field could highly improve the accumulation of the DOX@Gd-MFe_3_O_4_ NPs at tumor site. As a result, the MR signal at tumor region was strengthened due to the existence of large amount of DOX@Gd-MFe_3_O_4_ NPs. Moreover, T_1_ MR signal intensity was also measured at the tumor region at the determined intervals. As shown in Figure 7(C), MR signal of the two groups gradually increased to the peak at 4 h and then decayed over time, which was consistent to the MR images. Particularly, for the peak signal at 4 h, the magnet attached group was 1.6-fold compared to the group without magnet attachment. Furthermore, T_2_-weighted MR imaging was also performed at 4 h post-injection. For the control group, the tumor region exhibited no difference compared to muscle tissues. However, obvious tumor darkening effect was found at the tumor region upon the injection of DOX@Gd-MFe_3_O_4_ NPs (Figure 7(B)). Furthermore, in the presence of magnetic field, the tumor region became much darker compared to the group without external magnetic field. Consistently, T_2_ MR signals in tumors decreased to 0.46- and 0.26-folds for mice without and with a magnet attached, respectively compared to the control group (Figure S3). These results demonstrated the feasibility of DOX@Gd-MFe_3_O_4_ NPs for magnetic targeted dual T_1_/T_2_ MR imaging in specific cancer diagnosis.

### 3.9. *In vivo* anticancer outcome of combined PTT and chemotherapy

**Figure 8.**
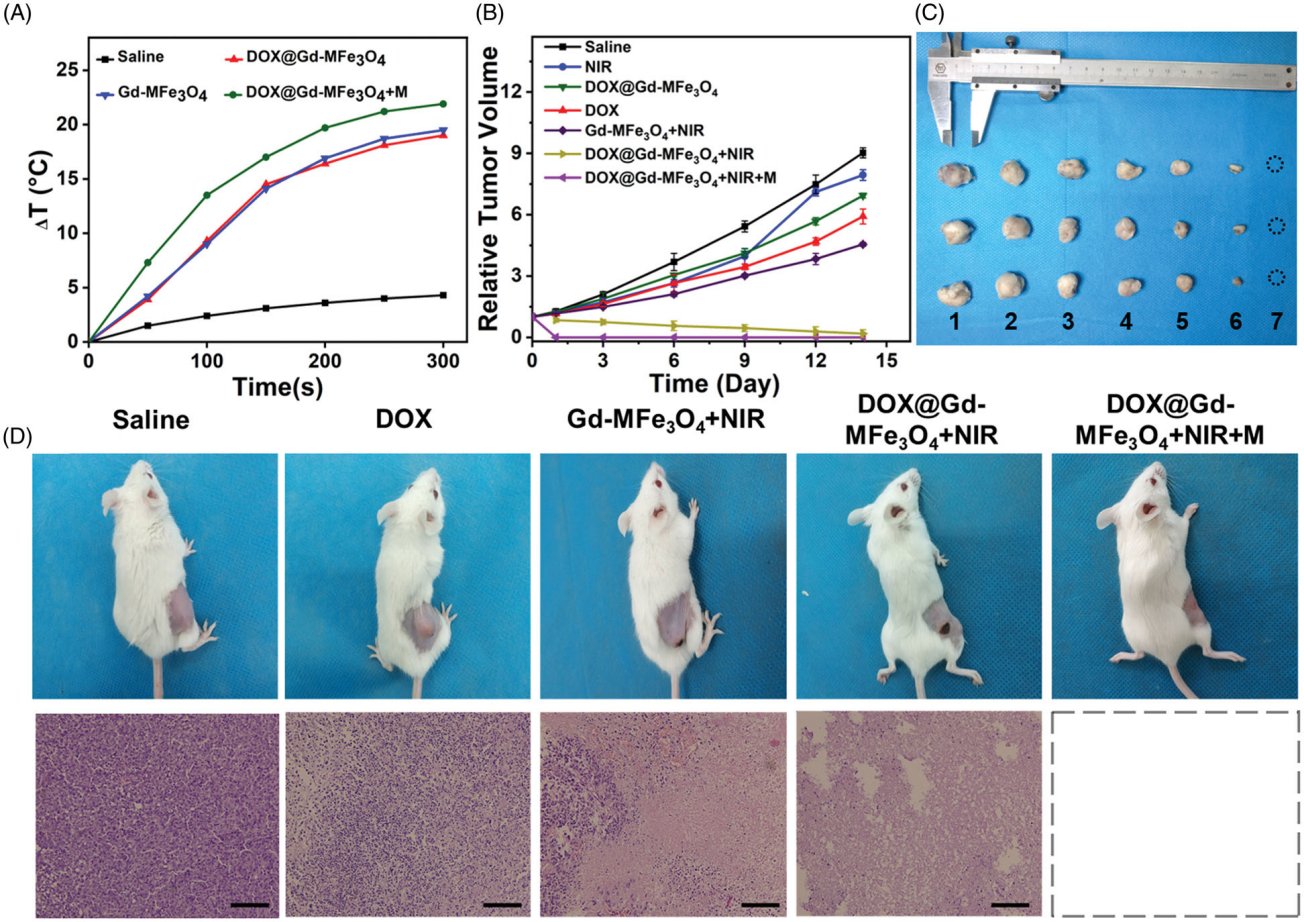
(A) Temperature changes in the tumor region under 808 nm laser irradiation at various injection; (B) relative tumor volumes of the 4T1 tumor bearing mice receiving various treatments for 14 days; (C) tumors excreted after 14-day with various treatments 1–7: saline, NIR, DOX@MFe_3_O_4_ NPs, DOX, MFe_3_O_4_ NPs + NIR, DOX@MFe_3_O_4_ NPs + NIR, DOX@MFe_3_O_4_ NPs + NIR + M; (D) Photographs of tumor bearing nude mice and the H&E staining of the tumor at 14 days for various groups, scale bar = 100 μm. Values are expressed as mean ± SD (*n* = 5).

Encouraged by the superior tumor therapeutic effect *in vitro*, we next evaluated the dual MR imaging-guided combined PTT and chemotherapy *in vivo*. The temperature elevation in tumor area upon NIR irradiation was monitored by an IR thermal camera, as shown in Figures 8(A) and Figure S4. For mice injected with saline, the tumor region merely increased by 4.1 °C under the NIR irradiation (1.5 W/cm^2^) for 5 min, suggesting the negligible effect of the NIR laser only. In contrast, for the mice receiving DOX@Gd-MFe_3_O_4_ NPs and MFe_3_O_4_ NPs, the laser induced rapid and significant temperature elevation at 17.5 and 18.2 °C, respectively, demonstrating the excellent ability of the prepared NPs to convert laser into thermal energy *in vivo*. In addition, as expected, when we attached a magnet at the tumor site, the mice revealed highest temperature increase of 22.7 °C at tumor area attributed to the magnetic field mediated tumor targeting.

Subsequently, the tumor volumes of each mouse after treatments were monitored for 14 days by a caliper. Afterwards, the result was presented as relative tumor volume compared to the initial volume, as shown in Figure 8(B). For the saline and NIR laser only treated mice, the tumor rapidly grew to 9.1- and 7.9-folds at the end, respectively, where the low temperature elevation at tumor site had limited tumor inhibition. On the other side, mice receiving free DOX, DOX@Gd-MFe_3_O_4_ NPs exhibited moderate tumor inhibition by 5.7- and 6.9-folds of initial volume, indicating that mono-chemotherapy could not efficiently suppress the growth of tumor in a long term. In contrast, the tumor growth was highly inhibited to 4.5-fold for Gd-MFe_3_O_4_ NPs-treated mice receiving NIR laser irradiation, demonstrating the effectiveness of mono-PTT in tumor inhibition. Furthermore, DOX@Gd-MFe_3_O_4_ NPs exhibited much higher tumor suppression to 0.4-fold under NIR laser irradiation. The therapeutic outcome was further enhanced when we applied external magnetic field to guide the accumulation of DOX@Gd-MFe_3_O_4_ NPs at the tumor site. In addition, mice and tumor photographs further verified the real therapeutic outcome of various treatments (Figure 8(C,D)). Moreover, no mice were found dead or body weight loss during the test period, revealing low effect of the formulations to mice (Figure S5). These results demonstrated the advantage of magnetic field guided combined PTT and chemotherapy in cancer treatment.

### 3.10. *In vivo* toxicity analysis

**Figure 9.**
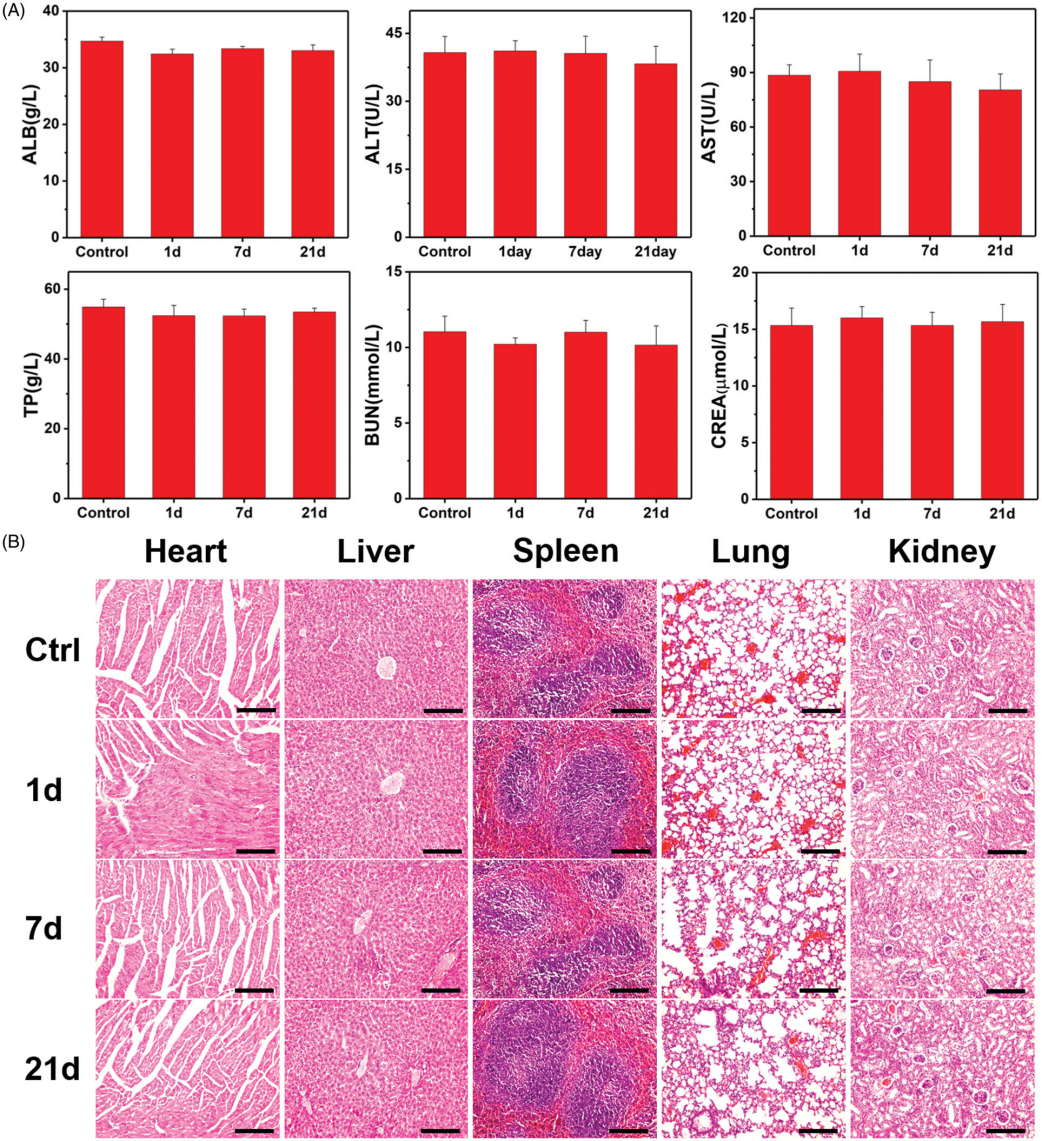
*In vivo* toxicity tests of Gd-MFe_3_O_4_ NPs: (A, a–f) mouse serum biochemistry analysis before (0d, control) and after injection of NPs for 1, 7, and 21 days. (B) Histological images of the heart, liver, spleen lung, and kidney of healthy mice 1, 7, and 21 days injected with Gd-MFe_3_O_4_ NPs and control mice. The organs were sectioned and stained with hematoxylin and eosin (H&E) and observed under a light microscope, scale bar = 100 μm.

The *in vivo* long-term biosafety was investigated on healthy Balb/c mice in a 21-day period. Gd-MFe_3_O_4_ NPs in saline were intravenously administrated in mice. Then, blood samples were collected from each mouse at 0, 1, 7, and 21 day post-injection for blood routine and biochemistry analysis. Several indicators were evaluated for the blood routine tests, including white blood cells (WBC), red blood cells (RBC), hemoglobin (HGB), platelet (PTL), mean corpuscular hemoglobin (MCH), mean corpuscular volume (MCV), mean corpuscular hemoglobin concentration (MCHC), and hematocrit (HCT). As show in Figure S6, all indicators in days 1, 7, and 21 did not reveal obvious variation compared with the control group in day 0. The serum biochemistry analysis was also conducted in the same condition. As revealed in Figure 9(A), the values of aspartate aminotransferase (AST), alanine aminotransferase (ALT), total protein (TP), indicators – albumin (ALB), blood urea nitrogen (BUN), and creatinine slightly fluctuated during the test period, where the values were still within the normal ranges for each indicator.

Furthermore, we also evaluated the biosafety of Gd-MFe_3_O_4_ NPs by the histological tissue analysis. Major organs were harvested after we withdrew blood samples, sliced, and stained with hematoxylin-eosin. As shown in Figure 9(B), the tissues in days 1, 7, and 21 revealed no obvious difference with the control group in day 0. Not only that, no remarkable inflammatory lesion and organ damage were observed in the experimental groups. These results confirmed the good biosafety of MFe_3_O_4_ NPs *in vivo* in a long term, which would be compatible for biomedical applications.

## 4. Conclusions

In summary, we successfully synthesized a magnetic targeted nanoplatform DOX@Gd-MFe_3_O_4_ NPs for T_1_/T_2_ dual modal MR imaging-guided synergistic cancer therapy. The prepared DOX@Gd-MFe_3_O_4_ NPs exhibited good colloidal dispersity, superior magnetic properties, superior NIR photothermal conversion, and NIR-triggered DOX release. High T_1_/T_2_ MR contrast was confirmed on the DOX@Gd-MFe_3_O_4_ NPs, which favored specific T_1_/T_2_ dual-modal MR imaging on 4T1 bearing mice *in vivo*. More than that, as a result of the good NIR photothermal conversion, DOX@Gd-MFe_3_O_4_ NPs demonstrated remarkably improved therapeutic effect of combined PTT and chemotherapy to eliminate cancer. Moreover, the blood and histological analysis validated the good biocompatibility of the NPs. Therefore, this versatile nanoplatform would provide a promising strategy for T_1_/T_2_ dual-modal MR imaging guided synergistic cancer therapy.

## Supplementary Material

Supplemental Material
