# Supplementary material for: Tumor-targeted Gd-doped mesoporous Fe3O4 nanoparticles for T1/T2 MR imaging guided synergistic cancer therapy
Source: Drug Deliv. 2021 Apr 19;28(1):787–99. doi: 10.1080/10717544.2021.1909177 (PMC8079076; doi:10.1080/10717544.2021.1909177)
Supplement: Supplemental Material [file IDRD_A_1909177_SM8485.docx]

Supplementary Data

**Tumor targeted Gd doped mesoporous Fe_3_O_4_ nanoparticles for T_1_/T_2_ MR imaging guided synergistic cancer therapy**

Shaohui Zheng^a,b,c,1,*^, Shang Jin^a,b,c,1^, Min Jiao^a^, Wenjun Wang^a^, Xiaoyu Zhou^a,b,c^, Jie Xu^a,b,c^, Yong Wang^a,b,c^, Peipei Dou^a^, Zhen Jin^d^, Changyu Wu^a,b,c^, Jingjing Li^a,b,c^, Kai Xu^a,b,c,*^

^a^ School of Medical Imaging, Xuzhou Medical University, Xuzhou, 221006, China

^b^ Department of Radiology, Affiliated Hospital of Xuzhou Medical University, Xuzhou, 221004, China

^c^Institute of Medical Imaging and Digital Medicine, Xuzhou Medical University, Xuzhou 221004, People’s Republic of China

^d^College of Medical Engineering, Xinxiang Key Laboratory of Neurobiosensor, Xinxiang Medical University, Xinxiang, Henan 453003, China.

^1^S. Zheng and S. Jin contributed equally to this work.

^*^Corresponding authors: [shaohui19910@163.com](mailto:shaohui19910@163.com), xkpaper@163.com

**Table. S1** Drug loading EE and LE of the DOX@ Gd-MFe_3_O_4_ NPs

| Composition | Encapsulation efficiency (%) | Drug loading efficiency (%) |
| --- | --- | --- |
| Gd-MFe_3_O_4_ :DOX(10:1) | 92.4±2.3 | 8.4±0.4 |
| Gd-MFe_3_O_4_ :DOX(10:2) | 63.5±3.1 | 10.5±1.1 |
| Gd-MFe_3_O_4_ :DOX(10:3) | 44.7±1.8 | 10.7±0.7 |

Values are expressed as mean±standard deviation (SD)(n=3)

Encapsulation efficiency (%) =$\frac{weight of DOX in NPs}{weight of feeding DOX}\times$100%

Drug loading efficiency (%) =$\frac{weigt of DOX in NPs}{weight of final NPs}\times$100%


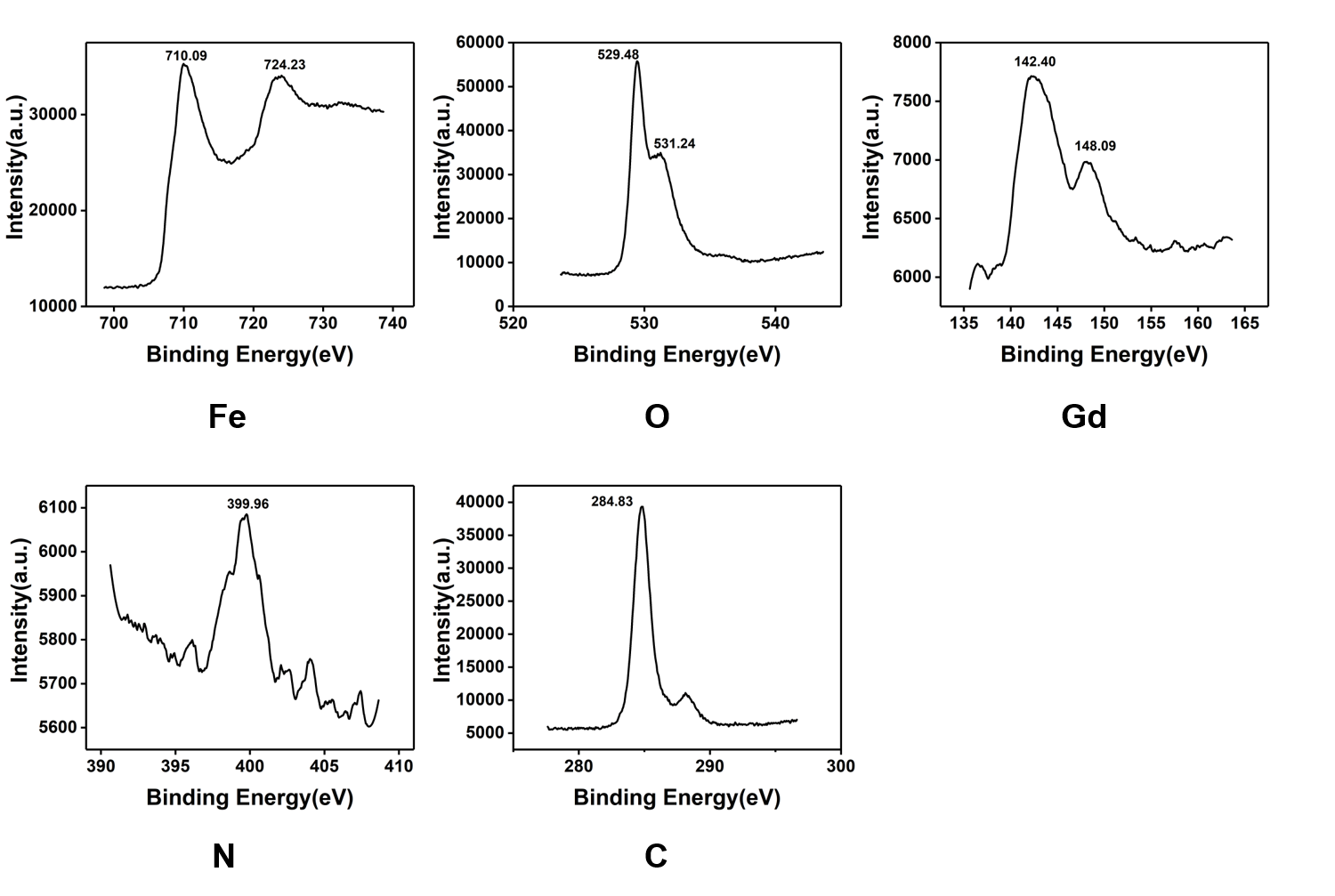


**Figure. S1** XPS analysis of Gd-MFe3O4 NPs for different elements: Fe, O, Gd, N, C.

**
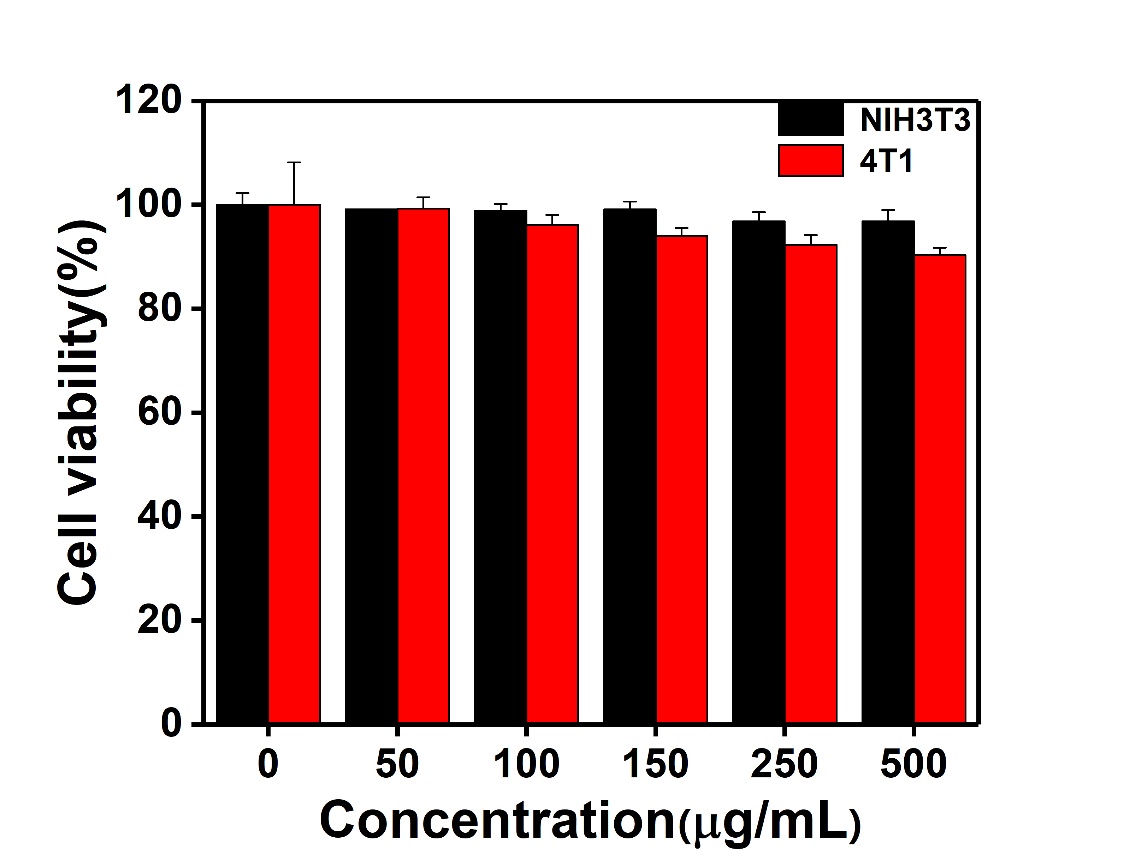
**

**Figure. S2** Biocompatibility test of Gd-MFe_3_O_4_ NPs against NIH3T3 and 4T1 cells.


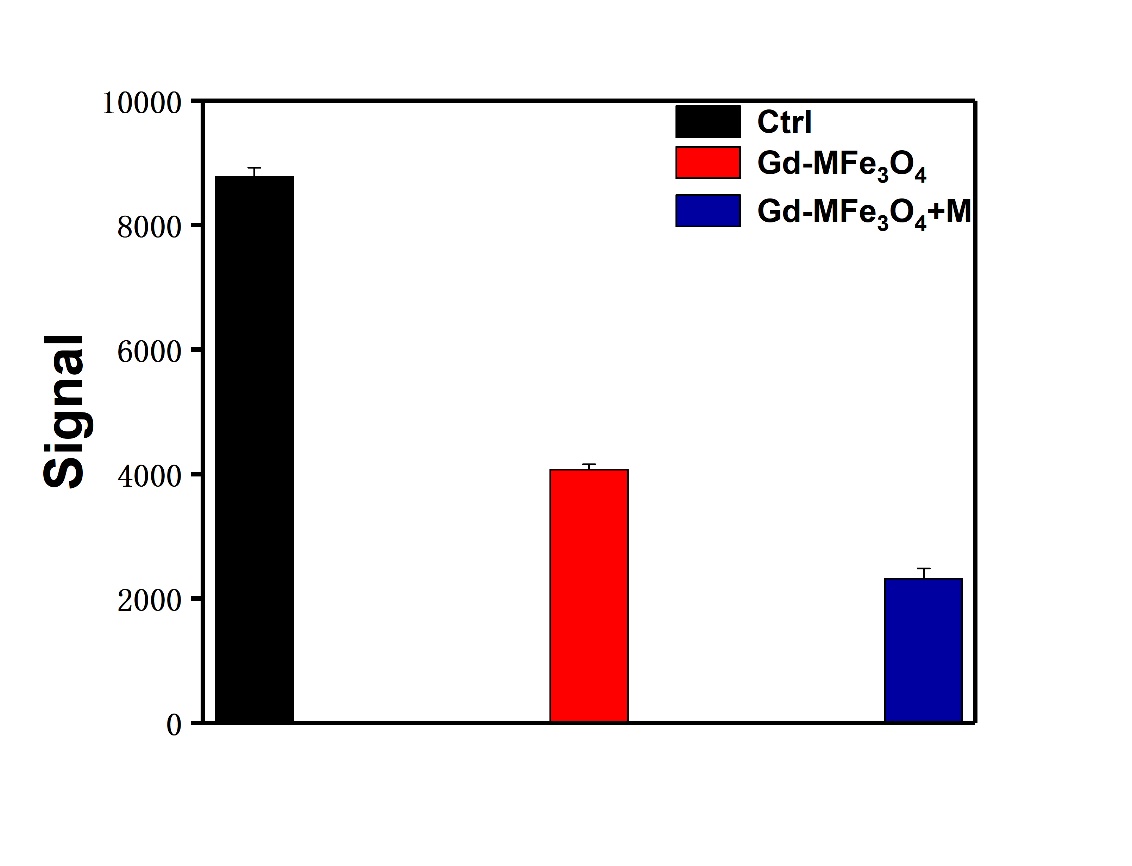


**Figure. S3** T_2_ signal of the tumor region at 4h post-injection of various NPs.

**
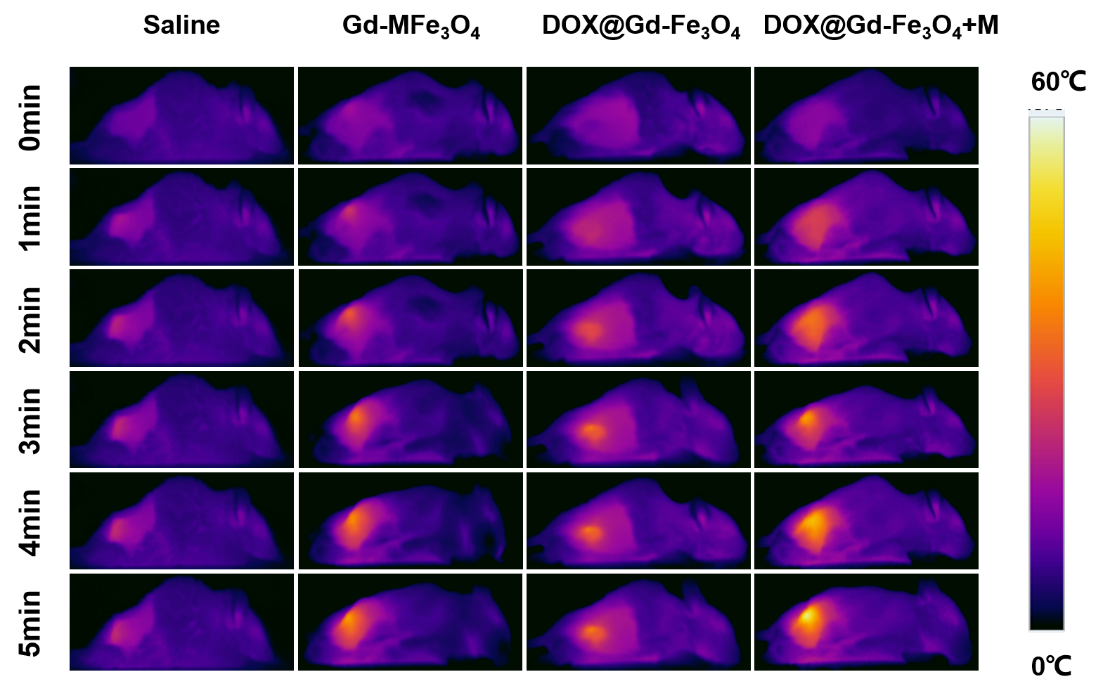
**

**Figure. S4** Thermal images of mice under NIR irradiation with various treatments.


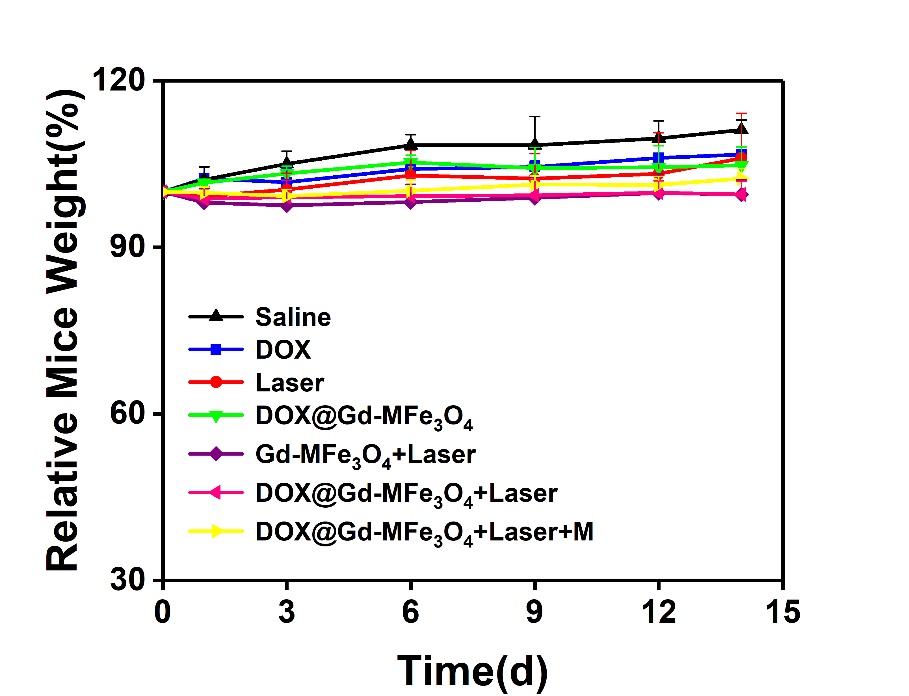


**Figure. S5** Relative mice body weight change after various treatments for 14 days.

**
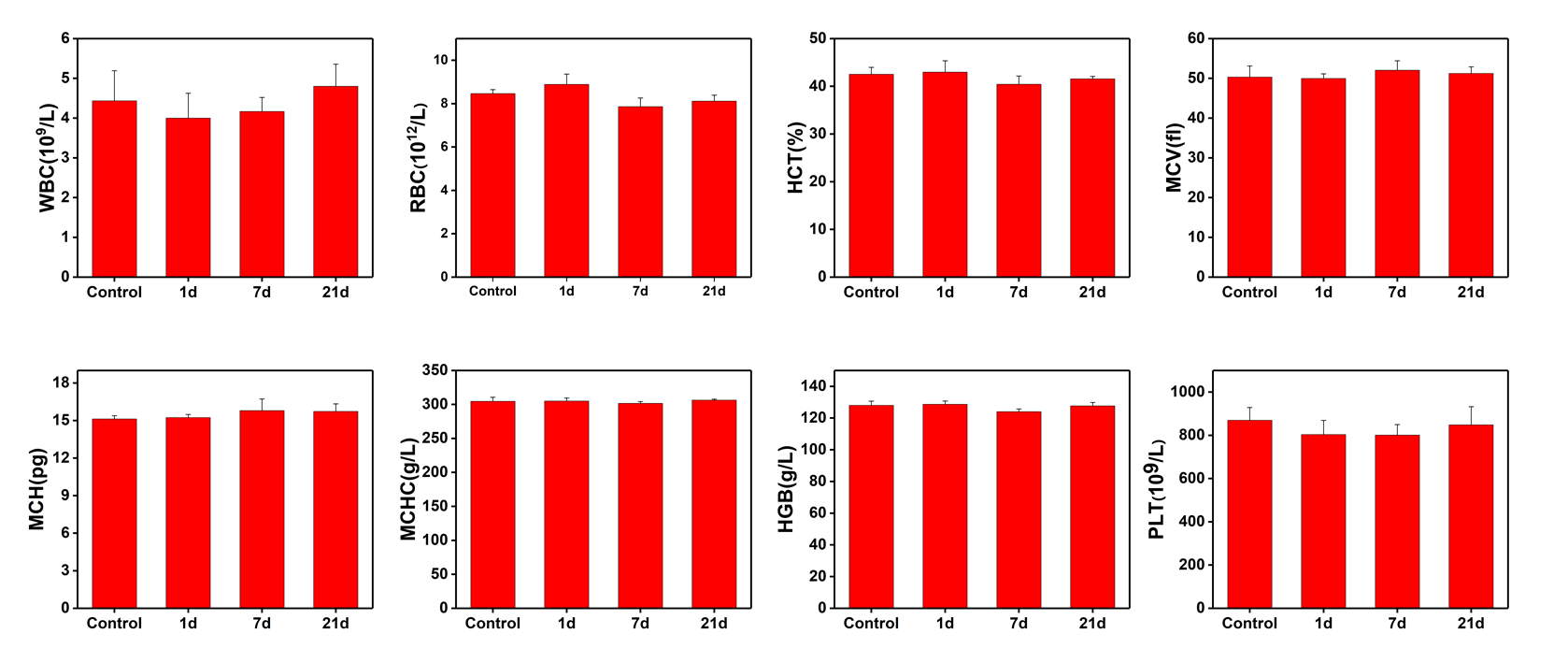
**

**Figure. S6** Blood routine analysis after injection of Gd-MFe_3_O_4_ NPs and saline for 0, 1, 7 and 21 days.


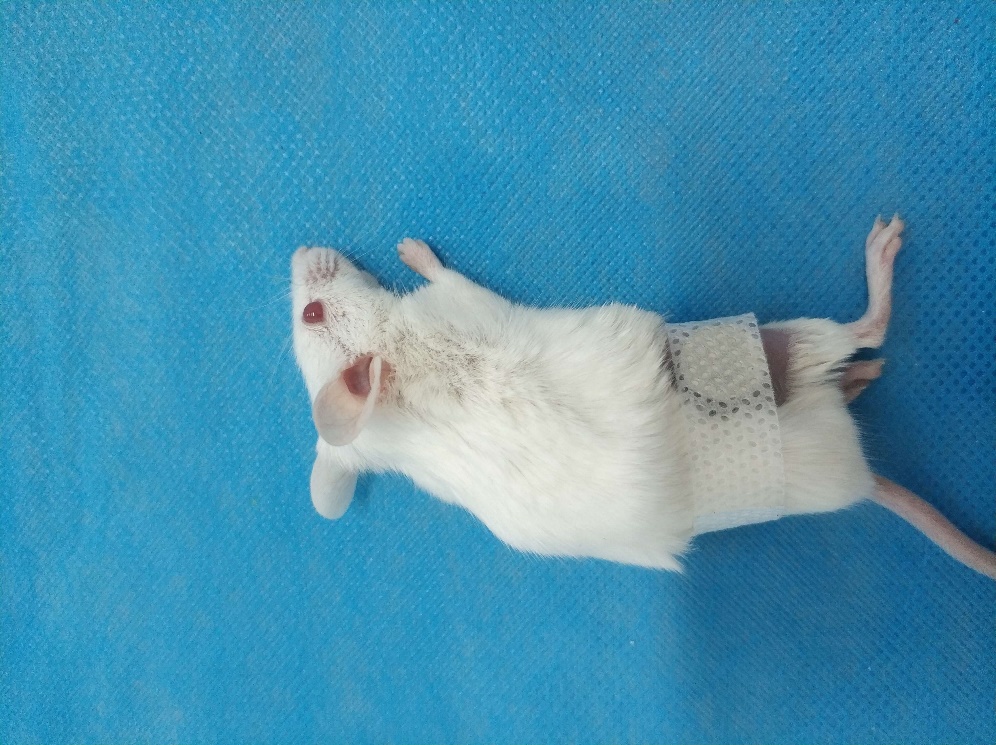


**Figure. S7** Mouse attached with a magnet using medical adhesive tape.

**Photothermal conversion efficiency calculation [Ref 1-2]**

The photothermal conversion efficiency (*η*) could be calculated using equation (1)

**** (1)

where *h*, *S* and *Q_diss_* are heat transfer coefficient, irradiated area and the baseline energy inputted by the sample cell, *T_max_* and *T_sur_* are the highest temperature of system and the temperature of surrounding, *I* and *A_808_* are the power density and absorption of NPs at 808nm respectively.

The value of *hS* is calculated by using the following equation (2) to (4):

 (2)

*$\tau_{s}=-ln\theta$* (3)

$\theta=\frac{T(t)-T_{sur}}{T_{\max}-T_{sur}}$ (4)

where *m* and *C_p_* are the mass of sample and the thermal capacity of sample and *t* is cooling time after irradiation.


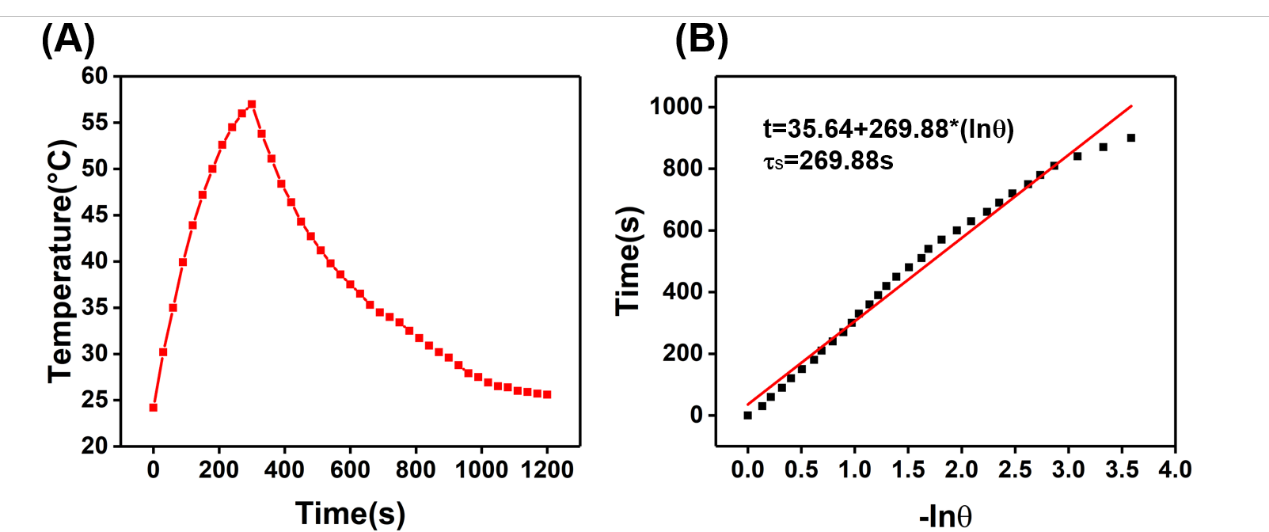


**Figure S8** (A) Heating/cooling experiment of 250 µg/mL DOX@Gd-MFe_3_O_4_ NPs aqueous solution under 1.8 W/cm^2^ 808 nm laser irradiation. The continuous wave laser was switched off after 5 min and the cooling rate was recorded. (B) Time constant for heat transfer from the system τ_s_, calculated by using the data after cooling period. The τ_s_ was determined to be 269.88s by applying the linear time data from the cooling period of versus negative natural logarithm of driving force temperature.

The result of that case irradiated under 808nm laser was that the value of *T_max_* - *T_sur_* equalled 32.5 °C, using the data of Fig. S8A. And the τ_s_ was 269.88 s, obtained by the negative natural logarithm of temperature (Fig. S8B). The value of *m* and *C_p_* were 1g and 4.2 J/(g·°C), respectively. Therefore, *hS* was calculated to be 15.56 mW/°C by using equation (2). *Q_diss_* and *A_808_* were measured independently as 30.21 mW with power (*I*) 1.8 W and 0.56 respectively. Substituting all of value to parameters into the equation (1), the photothermal conversion efficiency (*η*) of DOX@Gd-MFe_3_O_4_ NPs was calculated to be 26.8%.

**Reference:**

[Ref 1] Roper *et al*. Microscale heat transfer transduced by surface plasmon resonant gold nanoparticles. *J. Phys. Chem. C*, 2007, 111, 3636-3641.

[Ref 2] Feng *et al*. Programmed near-infrared light-responsive drug delivery system for combined magnetic tumor-targeting magnetic resonance imaging and chemo-phototherapy. *Acta Biomaterialia*, 2017, 49, 402-413.
